# SSA-ME Detection of cancer driver genes using mutual exclusivity by small subnetwork analysis

**DOI:** 10.1038/srep36257

**Published:** 2016-11-03

**Authors:** Sergio Pulido-Tamayo, Bram Weytjens, Dries De Maeyer, Kathleen Marchal

**Affiliations:** 1 Department of Information Technology, iGent Toren, Technologiepark 15, 9052 Gent, Belgium; 2 Department of Plant Biotechnology and Bioinformatics, UGent, Technologiepark 927, 9052 Gent, Belgium; 3 Bioinformatics Institute Ghent, Technologiepark 927, 9052 Gent, Belgium; 4 Dept. of Microbial and Molecular Systems, KU Leuven, Kasteelpark Arenberg 20, B-3001 Leuven, Belgium; 5 Grupo de Investigación en Ciencias Biológicas y Bioprocesos (Cibiop), Universidad EAFIT, Carrera 49 N° 7 Sur-50, Medellín, Colombia; 6 Department of Genetics, University of Pretoria, Hatfield Campus, Pretoria 0028, South Africa

## Abstract

Because of its clonal evolution a tumor rarely contains multiple genomic alterations in the same pathway as disrupting the pathway by one gene often is sufficient to confer the complete fitness advantage. As a result, many cancer driver genes display mutual exclusivity across tumors. However, searching for mutually exclusive gene sets requires analyzing all possible combinations of genes, leading to a problem which is typically too computationally complex to be solved without a stringent a priori filtering, restricting the mutations included in the analysis. To overcome this problem, we present SSA-ME, a network-based method to detect cancer driver genes based on independently scoring small subnetworks for mutual exclusivity using a reinforced learning approach. Because of the algorithmic efficiency, no stringent upfront filtering is required. Analysis of TCGA cancer datasets illustrates the added value of SSA-ME: well-known recurrently mutated but also rarely mutated drivers are prioritized. We show that using mutual exclusivity to detect cancer driver genes is complementary to state-of-the-art approaches. This framework, in which a large number of small subnetworks are being analyzed in order to solve a computationally complex problem (SSA), can be generically applied to any problem in which local neighborhoods in a network hold useful information.

Because of internationally coordinated efforts such as TCGA^1^,^2^ and ICGC^3^, a vast number of cancer datasets are publicly available. Using these datasets to identify mutations and pathways driving cancer phenotypes has become an active field of research^4^,^5^,^6^,^7^.

Efforts to search for driver genes in cancer tend to use single-gene tests, e.g. identification of significantly mutated genes based on background mutation rates (MutSitgCV^8^, MuSiC^9^), identification of genes which are enriched in mutations with high functional impact (Oncodrive-FM^4^) or identification of genes involved in tumorigenesis based on the spatial distribution of their mutations (somInaClust^10^). Most single-gene methods heavily rely on recurrent mutations in single genes across samples, thereby risking to miss rarely mutated genes.

Other methods do not perform their analysis at single gene level, but at the level of gene sets by exploiting the clonal properties of cancer. Tumorigenesis and tumor progression follow a clonal evolutionary model^11^,^12^,^13^,^14^. This has two consequences: first different tumors evolve independently. It has been shown that different tumors evolve by triggering the same driver pathways but not necessarily by affecting the same genes. Tumors thus display recurrent mutations at pathway level rather than at single gene level. A second property of the clonal evolutionary model is mutual exclusivity. In this view, the disruption of a single gene in a molecular pathway often yields the complete fitness advantage associated with disruption of that pathway, making additional mutations in the same pathway redundant^11^. This evolutionary property can be exploited to understand cancer mechanisms and identify driver mutations by searching for groups of genes that display mutual exclusivity with each other (i.e. groups of genes which have mostly one mutation per tumor).

A first series of methods that analyze gene sets assume that, because of the clonal properties of cancer cells, recurrent mutations should occur at the pathway level rather than at single gene level. These methods search for gene sets rather than single genes that display a certain property (high functional impact score, high frequency of mutations) and that are closely connected on an interaction network. This connectivity constraint reduces the search space in possible number of genes sets that have to be evaluated. As these methods (e.g. HotNet2^15^) rely on propagating information on an interaction network, they require information to be defined at the gene level (e.g. mutation frequency or gene scores).

A second series of methods make use of the mutual exclusivity property to analyze gene sets. They usually search for patterns of mutually exclusive genes (e.g. Dendrix^6^, MultiDendrix^16^ and CoMEt^17^). The identification of groups of genes showing mutual exclusivity across patients in large datasets has already been proven useful for the detection of driver mutations/pathways in single cancer types such as triple-negative breast cancer^18^, Lung Adenocarcinoma^16^ and in a pan-cancer setting^15^,^19^. Due to the combinatorics properties of the problem, these methods apply stringent upfront filtering to be able to analyze the data.

Some methods combine both clonal properties i.e. they search for mutual exclusivity and for recurrently mutated pathways (sets of mutually exclusive genes that tend to occur in pathways). However, because the ‘mutual exclusivity’ information can only be defined at the level of gene sets and not at the level of single genes, using the network does not sufficiently constrain the combinatorics of the problem. Because these methods have to analyze a large number of combinations of genes, the problem typically gets computationally too complex to be solved. Consequently, these methods use upfront filtering to reduce this computational complexity, thereby reducing the number of genes to analyze. Doing so, methods as MEMo^4^ and mutex^20^ filter upfront based on mutational frequency and are thus unable to take into account rarely mutated genes.

In order to provide a framework to assess mutual exclusivity while incorporating biological pathway information without the need for stringent upfront filtering, we developed SSA-ME (Small Subnetwork Analysis with reinforced learning to detect driver genes using Mutual Exclusivity). SSA-ME is a computational tool that searches for genes that show mutual exclusivity and that are closely connected on an interaction network to prioritize drivers. It uses a novel methodology named Small Subnetwork Analysis with reinforced learning (SSA) that divides a complex problem, i.e. finding driver genes that exhibit mutual exclusivity, into many simpler ones by calculating measures for mutual exclusivity in many small subnetworks. By solving these simpler problems iteratively, each time biasing the search space based on results of previous iterations, SSA-ME can prioritize potential driver genes with linear algorithmic complexity. This, in principle, allows it to process large input datasets in short computational times and therefore, in contrast to previous approaches, requires little upfront filtering.

To assess the performance of SSA-ME we analyzed each of the 12 TCGA Pan-Cancer tumor types^19^. Despite adding many more mutations to the input, we could prioritize well-known drivers that are found to be recurrently mutated in different tumors. However, in addition to prior findings we could prioritize several genes that displayed mutual exclusivity and pathway connectivity with well-known drivers, but that were rarely mutated in the different tumors and were missed by other methods that search for mutual exclusivity.

## Results

### SSA-ME Implementation

To identify cancer driver genes, we developed SSA-ME, a method that searches for small subnetworks of the interaction network containing mutated genes that show mutual exclusivity. SSA-ME approaches the complex problem of detecting driver genes by solving many independent and less complex sub-problems. In each sub-problem the method scores a set of genes which are close to each other in the interaction network for mutual exclusivity. SSA-ME scores many of these small subnetworks for their potential to contain genes exhibiting mutual exclusivity. Using these small subnetwork scores in a reinforced learning framework allows prioritizing individual genes that are likely involved in the cancer phenotype.

**Figure 1:**
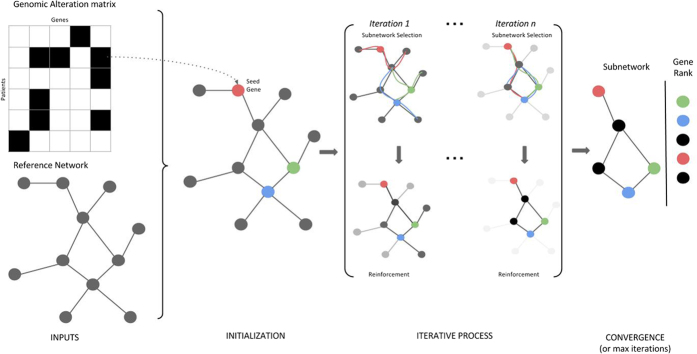
Overview of SSA-ME. The input consists of a matrix containing genomic alterations (i.e. mutations or copy number alterations, among others) across patients (depicted as black tiles) and a human reference network. In a first initialization step, every gene which has at least one genomic alteration across all patients is selected as a seed gene (colored genes in the network). The gene scores (represented as the opacity of the genes in the networks) are uniformly set to a value of 0.5. In every subsequent iteration step, small subnetworks will be generated, starting at every seed gene. Every gene adjacent to the small subnetwork has a chance proportional to its score to be incorporated in the small subnetwork. When a certain size has been reached the small subnetwork generation will stop and a score for each selected small subnetwork will be calculated based on the mutually exclusivity pattern found within this small subnetwork. At the end of every iteration step these small subnetwork scores will be used to update gene scores, altering the chance of genes to be incorporated into the small subnetwork in subsequent iteration steps. Upon convergence it can be seen that a few genes have high scores while others have scores close to 0. Genes are ranked based on their gene scores which reflects their potential to belong to a mutual exclusivity pattern.

The method is outlined in Fig. 1. SSA-ME searches the local neighborhood around a set of predefined seed genes. In this case, the seed genes correspond to all genes mutated in at least one sample. In each iteration step of the algorithm, genes in the neighborhood of a seed gene are selected into a small subnetwork with a chance proportional to their gene scores (which are chosen to be uniformly distributed in the first iteration). These small subnetworks are subsequently scored based on the mutual exclusivity signal of the genes in each small subnetwork. Individual gene scores are updated proportional to the mutual exclusivity scores of the selected small subnetworks to which they belonged. Updating of the gene scores modifies the likelihood with which each gene will be selected in subsequent iteration steps. The iterative process continues until the method converges to a solution or a maximum number of iterations is reached. The output of SSA-ME consists of a ranked list of prioritized potential drivers supported by bootstrap and an interactive network visualizing the prioritized drivers together with supporting files compatible with Cytoscape^21^.

### Performance on simulated data

To evaluate the robustness of the method with respect to the used reference network, we applied SSA-ME on a simulated dataset in combination with a high quality human reference network and underconnected/overconnected versions of this reference network (with respectively 10%, 25% and 50% of the network edges being deleted or added). Per network, 100 simulations were performed. Each simulated dataset contained a target gene set of mutually exclusive genes consisting of maximally 20 genes that are connected on the reference network and that were mutated in 30% of the samples (see Materials and Methods).

**Figure 2:**
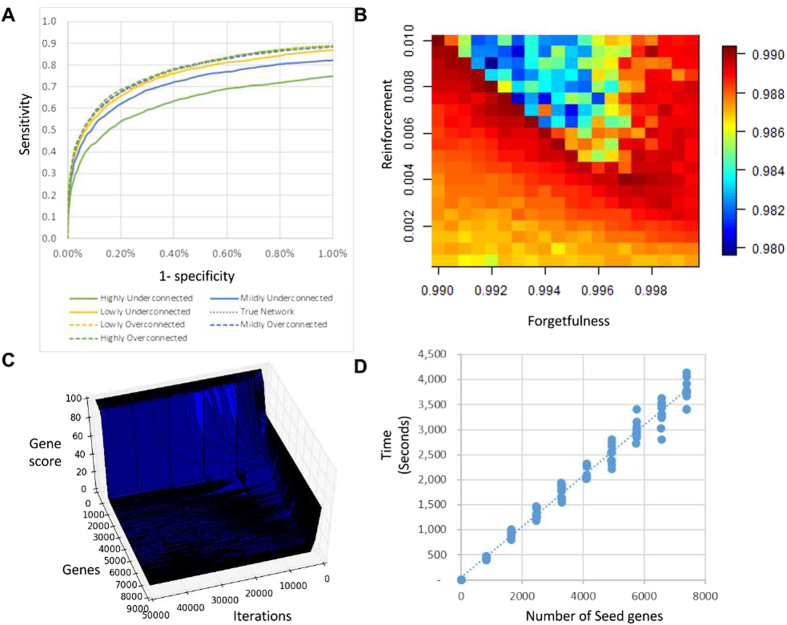
Performance on Simulated Data. (**A**) Robustness of the predictions with respect to the used reference network. The X-axis represents 1-specificity and the Y-axis represents sensitivity (ROC curve). Underconnected networks lead to lower performance while overconnected networks result in similar, although lower, performance as compared to the performances obtained with the original network. Note that, for clarity reasons, the range of the x-axis is restricted to [0, 0.01]. (**B**) Heat map depicting parameter sensitivity. Area under the ROC curve (AUC) values for every analyzed parameter pair are depicted. Warm colors depict higher AUC values while cold colors depict lower AUC values. It can be seen that the best performance is achieved on the diagonal for combinations of reinforcement and forgetfulness of 1. (**C**) Plot visualizing convergence and stability of convergence of gene scores. The X-axis represents the number of performed iterations, the Y-axis displays all genes in the reference network (black lines in the plot) and the Z-axis represents the gene scores. All genes start on the right side with a gene score of 0.5. Most of them converge fast to 0 or 1. As no inflecting lines are observed, convergence is stable. Results are shown on a plot depicting scores for all genes at every iteration step. (**D**) Plot showing linear time complexity of the algorithm with respect to the number of seed genes. Each dot on the plot represents the time to convergence of a separate run. Per tested number of seed genes, 10 simulations were performed. Results were obtained by running the algorithm on one single processor Intel(R) Xeon(R) CPU E5-2670 0 @ 2.60 GHz.

Applying SSA-ME on each simulated dataset resulted in a ranked gene list. The top x% of the gene list were considered as driver genes. Performance was evaluated by plotting the sensitivity versus the specificity where the sensitivity is defined as the percentage of genes belonging to the target gene set that was retrieved amongst the x% highest ranked genes and the specificity is defined as the proportion of genes not present in the target gene set that were correctly classified as non-drivers. The results are shown in Fig. 2A for the highest ranked genes as this is the range that is of biological relevance (correctly identifying positives). The full ROC plot and the sensitivity/PPV plots can be found in Supplementary Fig. 1.

Figure 2A indicates that the best performance is obtained using the reference network without added or deleted edges, as for the same relative increase in sensitivity less false positives are predicted (lower relative increase in 1-sensitivity). The method shows in general a high resilience of the results to using an overconnected network. In this case the method is capable of successfully prioritizing most of the genes in the mutually exclusive gene set with a low number of false positives (which is the range we envisage when only showing the values of the 1-specificty between 0 and 0.01). With an underconnected network the maximal sensitivity that can be reached will get restricted as some of the genes that show mutual exclusivity can no longer be connected in the network.

To assess the sensitivity of the method versus its parameter settings we ran SSA-ME on the same simulated data each time using a different combination of the reinforcement and forgetfulness parameters. Reinforcement determines the maximal value by which a gene score can be increased in the next iteration. Forgetfulness determines the fraction of the gene score that is retained in each subsequent iteration. Hereby reinforcement values were varied from 0.0005 to 0.0100 in steps of 0.0005. Forgetfulness values varied from 0.99 to 0.9995 in steps of 0.0005. Note that values of the forgetfulness closer to 1 imply that less is ‘forgotten’ and values of reinforcement are consistently lower than the ones of the forgetfulness to ensure that only true positives will be reinforced. For each parameter combination 10 simulated datasets were analyzed. The performance per parameter combination was assessed using the mean value of the area under the ROC curve (Fig. 2B). In general, a low performance is obtained if the forgetfulness is relatively low compared to the reinforcement. In those settings false positives might become reinforced relatively more than some weak or isolated true positives. However, when the forgetfulness is close to 1, the performance is more robust to the choice of the reinforcement value. Alternatively, when the forgetfulness is too high compared to the reinforcement, true positives retain too little gene score which results in a more random selection of nodes, hence incorporating more false positives. Best performances were obtained on the diagonal where the sum of the values of *r* and *f* is close to one: *r* + *f* ≈ 1. In most cases, a combination where the sum of the reinforcement and the forgetfulness is higher than one results in lower performances because then again the reinforcement becomes relatively high compared to the forgetfulness, resulting in relatively more false positives.

To show that the method converges to a stable solution, we ran it on one simulated dataset for 50.000 iterations. Figure 2C shows that the method exhibits a consistent behavior, i.e. after a gene obtains a high gene score, it will remain consistently high or vice versa. Furthermore, this figure shows that the algorithm converges, provided a sufficient number of iterations have been performed.

To analyze its complexity with respect to the number of seed genes, we ran SSA-ME on 10 different simulated datasets, each time using an increasing number of seed genes (ranging from 1 to 8000 genes). Datasets contained incrementally more added seed genes. Seed genes were added gradually according to the frequency with which they were found mutated in the different tumor samples, hereby assuming that the most frequently mutated genes are the ones that in a real setting would also be prioritized as the most promising seeds. These runs were repeated on 10 different simulated datasets. Results are visualized in Fig. 2D and clearly show the linear complexity of the algorithm with respect to the number of seed genes.

### Analysis of TCGA data

To test the biological relevance of SSA-ME, we applied it to each of the Pan-Cancer TCGA cancer datasets^19^. In this section we primarily focus on the well-studied Breast cancer dataset as a benchmark but also show the most interesting results of the Pan-Cancer analysis. All remaining Pan-Cancer TCGA results can be found in Supplementary materials.

**Figure 3:**
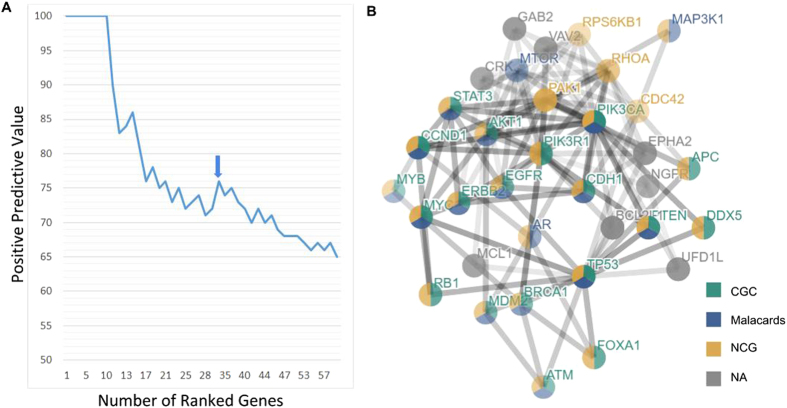
Application of SSA-ME on TGCA Breast Cancer dataset. (**A**) Determination of the number of genes to be prioritized as cancer drivers. Genes were ranked according to their gene score obtained by SSA-ME. The X-axis represents the number of genes in the list of prioritized genes obtained by setting a cut-off on the rank. The Y-axis represents the positive predictive value (PPV) for the genes present in each list that corresponds to a given rank threshold. The PPV is defined as the number of true driver genes prioritized divided by the number of prioritized genes. Note that the true driver genes are defined as all genes present in CGC. At the chosen threshold (arrow) 34 potential cancer drivers were prioritized. (**B**) Subnetwork obtained after using SSA-ME on the TGCA breast cancer dataset. Genes are represented by nodes. If the gene had been associated with cancer, this is indicated by the color of the database in which the association was described. Gray genes correspond to genes not present in the Census of Cancer Genes, Malacards (breast cancer) or the Network of Cancer Genes database. The size of the node reflects the number of samples in which a gene was found mutated.

For the analysis we used a high quality human interaction network (see Materials and Methods). As seed genes we used all genes carrying at least one somatic mutation or copy number alteration in any of the samples. After running SSA-ME, genes were prioritized as putative drivers based on their ranks by using a cut-off on the ranked list. This cut-off was chosen to provide a good trade-off between sensitivity and precision (i.e. an adequate positive predictive value (PPV) based on the genes present in the Cancer Gene Census (CGC)^22^ as true positives) (Fig. 3A). Note that the PPV represents a lower boundary on the actual number of true positive predictions as all genes not present in the CGC are regarded as false positives. This is particularly true in this analysis because CGC defines “known” cancer genes merely based on their somatic mutational load: This excludes genes implicated in cancer based on expression values, epigenetics, germline variants and amplifications/deletions if it is deemed that the amplification/deletion cannot be attributed to a single or a few genes with a sufficient amount of evidence^22^.

In the breast cancer dataset, we identified 34 potential driver genes. Figure 3B displays these genes in the form of an interaction network where the nodes are genes and the edges are interactions connecting them. Because of the nature of the method this prioritized gene list contains putative drivers, but also ‘linker genes’ that connect genes showing mutual exclusivity but that are not mutated themselves in any of the breast cancer samples. These ‘linker genes’ are therefore not drivers within the available tumor samples, but have driver potential as they were found to connect drivers through the network.

Most of the prioritized genes (26 out of 34) have previously been mentioned in catalogues of genes implicated in cancer (CGC, NCG or the most relevant Malacard) (Supplementary Table 1). 2 genes of 26 (*CDC42* and *BCL2L1*) were selected as ‘linker genes’ (i.e. did not display alterations in the breast cancer dataset). *CDC42* is a candidate cancer driver according to NCG and is also listed in the “Breast cancer” malacard. *BCL2L1* is mainly associated with colorectal cancer and lung cancer^23^,^24^,^25^ through gene expression changes and is also selected as a driver mutation in other TCGA datasets (see below). This confirms the driver potential of the identified linker genes. Amongst the prioritized genes, 9 are rarely altered (in <1% of the samples, at most 10 alterations in the breast cancer dataset, i.e. *BCL2L1*, *CDC42*, DDX5, AKT1*, VAV2*, *EPHA2*, *CRK*, *UFD1L*, *NGFR* and *APC*), indicating our method is able to also prioritize genes with few genomic alterations. For genes with such low mutational load it is impossible to statistically or visually prove mutual exclusivity. These rarely mutated genes are retrieved by SSA-ME, despite having few mutations, when they exhibit at least partial mutual exclusivity with the surrounding genes in the network. If these surrounding genes exhibit sufficient mutual exclusivity with each other, the rarely mutated gene is selected based on its association with that pattern of mutual exclusivity. The fact that of the 10 rarely mutated genes, 5 (BCL2L1, CDC42, *DDX5, AKT1 and APC*) are listed in cancer gene databases indicates such association-based selection is useful.

**Figure 4:**
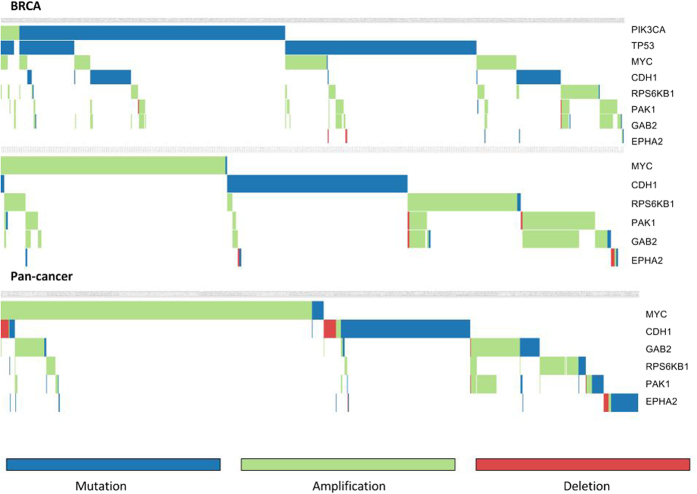
Mutual exclusivity pattern for *EPHA2*. Green tiles depict copy number gains, orange tiles depict somatic mutations and red tiles depict losses of copy number. The top figure is the mutual exclusivity pattern for *EPHA2* in the breast cancer dataset. The middle figure is the same pattern but with *PIK3CA* and *TP53* left out in order to allow zooming in on the least frequently mutated genes. The bottom figure provides the pan-cancer view of the pattern detected in breast cancer (also with PIK3CA and TP53 left out). Patterns were created with Gitools^41^.

To uncover the driving force behind the selection of the prioritized genes, the five small subnetworks with the highest mutual exclusivity scores (see materials and methods) were retained for each prioritized gene. As an illustrative example the mutual exclusivity pattern of the union of these networks is shown for *EPHA2*, one of the prioritized genes that was rarely mutated in breast cancer and not listed in any of the used reference cancer databases. The EphA2 receptor is involved in multiple cross-talks with other cellular networks including EGFR, FAK and VEGF pathways, with which it collaborates to stimulate cell migration, invasion and metastasis^26^. We did prioritize *EPHA2* as a driver in breast cancer, despite its relatively low number of mutations. This because it showed (near) perfect mutual exclusivity with the well-known drivers *PIK3CA*, *GAB2, PAK1* and *RPS6KB* and all members of the PI3K pathway known to act downstream of *EPHA2*. These results were confirmed by the visualization of the mutual exclusivity patterns at pan-cancer level (Fig. 4). The clear mutual exclusivity of *EPHA2* with the aforementioned genes at pan-cancer level are mainly due to the contribution of the Head and Neck squamous cell carcinoma tumor samples (HNSC) in which *EPHA2* was found to be more frequently mutated. Consistently, *EPHA2* was also highly prioritized by our analysis of the HNSC dataset (see supplementary Notes). The illustrative example shown in Fig. 4 also demonstrates that although SSA-ME is not designed to retrieve the largest mutual exclusive subnetwork, the selected small subnetworks that drive the selection of the prioritized genes do show mutual exclusivity. Note that *PAK1* and *GAB* are by definition not mutually exclusive as they belong to the same amplicon (see supplementary Notes).

**Figure 5:**
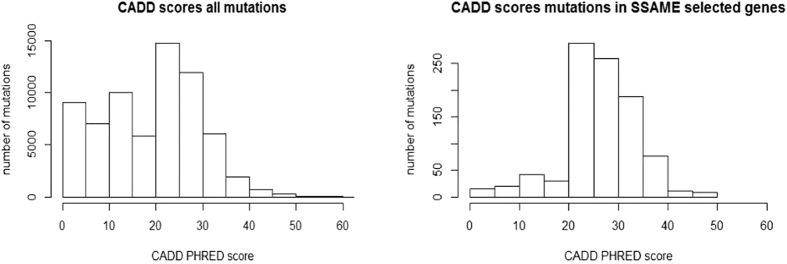
Analysis of selected genes. CADD score distribution of all mutations (left histogram), and of the set containing the mutations in the genes prioritized by SSA-ME (right histogram). The X-axis depicts the CADD score and the Y-axis depicts the frequency of mutations having a CADD score within a certain range.

Figure 5 shows that the somatic mutations carried by the 34 prioritized genes follow a CADD^27^ score distribution significantly higher (Wilcoxon rank sum test, W = 44197000, p = 2.2 × 10^−16^) than the CADD score distribution of all present somatic mutations, pointing towards the functional relevance of at least some of the mutations carried by the predicted drivers. Of the 34 ranked genes, 10 genes were not listed in cancer gene databases (*VAV2*, *EPHA2*, *BCL2L1*, *CRK*, *GAB2*, *TPS6KB1*, *UFD1L*, *NGFR*, *MCL1* and *PAK1*) based on CGC version 77, NCG 5.0 or the Malacards Breast Cancer category version 1.11.724. To further investigate these putative cancer drivers, we compared the distributions of the mutual exclusivity scores of the small subnetworks derived from respectively the real and randomized data to which the putative driver genes belonged (Supplementary Fig. 2). These results indicate that the mutual exclusivity scores of the subnetworks from which the prioritized genes were derived were always significantly higher in the real than in the randomized data, even when accounting for the fact that the mutual exclusivity scores decrease globally when using randomized data (Supplementary Table 2).

We also ran SSA-ME on the remaining 11 Pan-cancer datasets (Supplementary Figs 3–13; Supplementary Tables 3–13). In order to identify promising candidate driver genes, we identified the genes that were recurrently prioritized as driver genes in different Pan-cancer datasets. Interesting prioritized genes include *VCAN* (identified in STAD, LUAD, BLCA and fell just out of PPV cutoff in UCEC), *UBE2I* (identified in OV, STAD and fell just out of PPV cutoff in HNSC) and *BCL2L1* (identified in OV, BLCA, COADREAD, LUAD, UCEC and LUSC). *BCL2L1* was selected in 7 out of 12 analyzed cancers. While it was selected as a linker gene in BRCA, it has primarily gain of copy numbers in OV, BLCA, COADREAD, LUAD, UCEC and LUSC. Further literature-based evidence for the most interesting putative driver genes from the BRCA dataset and the PAN-cancer dataset can be found in Supplementary Notes.

### Comparison with other methods

To compare SSA-ME to other methods, we obtained the results of MutSigCV^8^, MutSig2CV^28^, Mutex^20^ and Oncodrive-FM^5^ when run on the TCGA Breast cancer data. MutSigCV^8^, MutSig2CV are representatives of single-gene prioritization methods that test whether a gene is mutated more than expected by chance. Oncodrive-FM prioritizes by searching for genes that are enriched in mutations with a high functional impact. Mutex searches for mutual exclusivity modules using a reference network.

**Figure 6:**
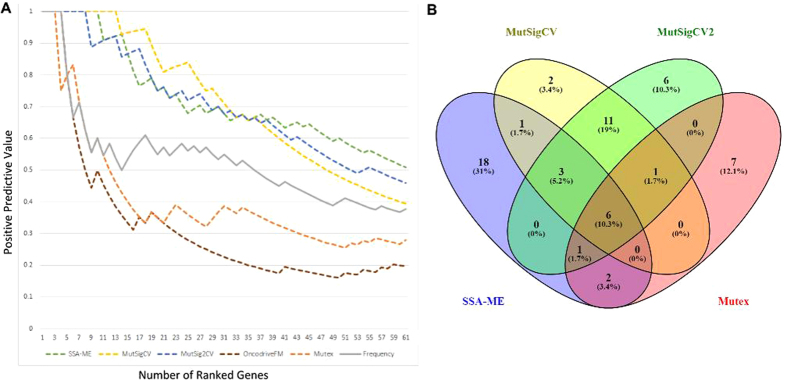
Comparison between SSA-ME and related methods. (**A**) The positive predictive value (PPV) of the results of multiple methods when analyzing the breast cancer dataset. The PPV is defined as the number of true driver genes prioritized divided by the number of prioritized genes. Note that the true driver genes are defined as all genes mentioned in CGC. (**B**) Overlap of prioritized driver genes between the different methods. Venn diagram created with VENNY^42^.

We used the positive predictive value using genes mentioned in CGC as true positives to compare the performance of the different methods to each other. These results are depicted in Fig. 6A. From this it can be seen that SSA-ME performs about equally well as its competitors given the evaluation criteria. In all cases the methods will be penalized for finding relevant novel predictions not present in CGC. As the known drivers might be biased towards unknown properties (e.g. mutational recurrence) it is hard to predict which methods will be affected most by false negatives in CGC. The relative under/over performance of certain methods over other methods should therefore be interpreted with care.

Figure 6B shows to what extent the different methods prioritize the same genes. For each method we selected from the top 61 ranked genes (61 is the number of genes ranked by SSA-ME after bootstrapping on the top 100 ranked genes prior to bootstrapping) only those present in CGC and we show their overlap. We used genes present in CGC to reduce the number of false positives for this analysis. As expected, the more similar the concept of two methods, the more similar their results. The single gene methods MutSigCV and Mutsig2CV are comparable and SSA-ME shows the highest relative overlap with Mutex as both methods are network-based and use mutual exclusivity. However, in general SSA-ME selects several known cancer genes that were not selected by any of the other methods (58% of genes selected by SSA-ME), indicating the complementary of SSA-ME to the other methods in selecting drivers. The complementarity with single gene methods is understandable given that SSA-ME uses different properties (mutual exclusivity of a gene set rather than frequency-based properties of single genes). Part of the difference between SSA-ME and Mutex can be explained by the difference in filtering (we can find more genes as we do not need to apply a stringent criteria). Remaining differences might relate also to the fact that Mutex uses, as an integral part of the method, a directed signaling network different from the interaction network used by SSA-ME. Note that the genes selected by SSA-ME show a very low number of mutations in some genes. For example, of the 18 genes selected only by SSA-ME, 7 genes contained less than five mutations compared to just one of the 19 genes selected only by MutSig2CV and MutSigCV together. This indicates that SSA-ME is complementary to the other methods in finding rarely mutated driver genes.

A widely used method that is conceptually most similar to SSA-ME is MEMo as it also uses mutual exclusivity over an interaction network. However, we were not able to run MEMo on the used datasets so we could not directly include it in the comparison described above. In order to compare the results with MEMo, we ran SSA-ME on the 2012 TCGA BRCA data using the same criteria to filter the input data as was used in the original MEMo publication. It can be shown that we are able to find largely the same results in this case. The main advantage of SSA-ME compared to MEMo is that SSA-ME can be run on larger, much less stringently upfront filtered, datasets. The result of this comparison is in Supplementary Notes.

## Discussion

We introduce SSA-ME, a tool for prioritizing cancer driver genes using mutual exclusivity with SSA (Small Subnetwork Analysis). SSA is a small subnetwork analysis technique with reinforced learning which solves a complex combinatorial search problem over an interaction network by calculating, in this case, measures for mutual exclusivity in many small subnetworks. The framework can be generically applied to any problem in which local neighborhoods in a network hold useful information.

Here we applied SSA to prioritize cancer driver genes that are in each other’s neighborhood in the interaction network and at the same time display mutual exclusivity across different tumor samples (referred to as SSA-ME). To overcome the inherent high algorithmic complexity posed by its combinatorial nature, the problem of identifying drivers is iteratively solved and in each iteration multiple small subnetworks are independently analyzed for mutual exclusivity. All results of these small subnetwork analyses are used in subsequent steps to bias the search space. The advantage of splitting the complex problem into multiple less complex problems, is that SSA-ME is not restricted by the number of mutated genes in the input data. By circumventing the stringent filtering strategy that is required by most other methods to evaluate mutual exclusivity, SSA-ME can identify drivers that are rarely mutated. These mutations are normally lost when an upfront filtering is used based on the mutation frequency across samples.

When prioritizing drivers by searching for closely connected genes on an interaction network that exhibit mutual exclusivity, the incompleteness of the interaction network might lead to an underestimation of the number of potential driver genes. Missing edges in the interaction network could refrain the method from connecting some driver genes. Given we search for small subnetworks, our method shows resilience towards incomplete or underconnected networks as was shown by the simulated data and was able to find drivers even when mutual exclusivity had been heavily disrupted.

The search for small subnetworks comes at the expense of never explicitly searching for the largest patterns of mutual exclusivity. Such largest patterns of mutual exclusivity can only be approximated by merging small subnetworks with high scores to which a prioritized gene belongs. They provide a good approximation but there is no guarantee that *all* genes within such pattern are mutual exclusive with each other.

The performance of SSA-ME in terms of a positive predictive value based on known genes associated with cancer was comparable to widely used methods. An important observation is the fact that, while the SSA-ME and the other driver identification methods share some findings, SSA-ME was also able to prioritize a large number of genes not found by any other method, indicating the complementarity between SSA-ME and the other methods. In contrast to the single-gene methods, SSA-ME relies also on the use of the interaction network and mutual exclusivity. As compared to MEMo and Mutex, which use an interaction network and mutual exclusivity, SSA-ME is the only method that can deal with a large number of input mutations and is therefore able to use mutual exclusivity to drive the gene prioritization.

## Materials and Methods

### SSA-ME

**Figure 7:**
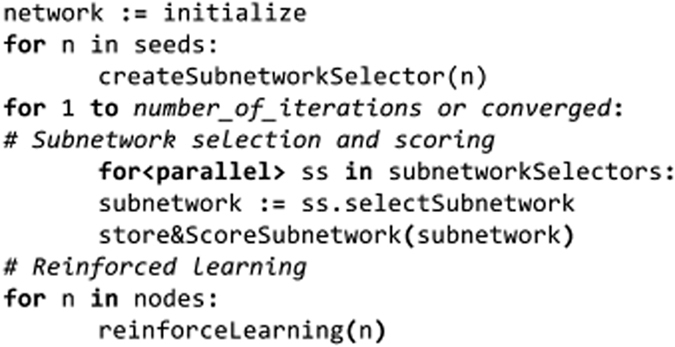
Pseudocode of SSA-ME algorithm.

Small Subnetwork Analysis with reinforced learning to detect driver genes using Mutual Exclusivity (SSA-ME) is an algorithm that uses an interaction network to detect driver genes by exploiting mutual exclusivity in cancer. To accomplish this, SSA-ME performs two independent functions in an iterative manner: small subnetwork selection/scoring and reinforced learning. Each gene (node) in the interaction network is initialized with a uniform gene score. Then, iteratively: starting from a set of seed genes, small subnetworks are selected favoring genes with high gene scores. Each selected small subnetwork is then scored based on how well the genes composing the small subnetwork exhibit mutual exclusivity. Genes that consistently belong to small subnetworks with high mutually exclusivity scores are more likely to be selected in subsequent iterations. This will lead to high gene scores for genes which are involved in local gene sets showing mutual exclusivity, and therefore are possible drivers. The pseudocode describing the algorithm can be found in Fig. 7.

#### Initialization

The algorithm is initialized by giving each gene (node) an initial gene score of 0.5. A static list of seed genes is defined that contains genes which are possibly driver mutations. Any type of biologically relevant filtering can be used to generate such gene list. In the context of this paper, seed genes are defined as all genes that were found to be altered in at least one sample (tumor).

#### Small subnetwork selection and scoring

Within each iteration step small subnetworks of equal size are selected. Starting from every seed gene, subnetworks are selected by subsequently adding a gene which is connected to the current subnetwork, expressing the assumption that mutually exclusive genes are likely to be located in the same adaptive pathway. In order to evaluate gene sets of different sizes for mutual exclusivity, the size of the small subnetworks varies from 3 to 6 genes between iterations. The probability of adding a gene to a small subnetwork is proportional to the gene scores of genes connected to the small subnetwork. Once constructed, each small subnetwork receives a mutual exclusivity score (MES). Each sample (tumor) contributes to this score with a weight that is inversely related to the number of genes from the small subnetwork that were found mutated in that sample. This is calculated using the following equation:


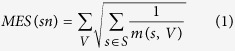


where *V* are the genes present in small subnetwork *sn* ordered according to the number of samples in which these genes were found to be mutated. *S* is the set of samples pending to contribute to the mutual exclusivity score. Initially *S* includes every sample with a mutation in one of the genes in the small subnetwork, but every time a sample is used to calculate a mutual exclusivity score it is removed from *S*. In this way a sample can only contribute once to the *MES. m(s*, *V*) is the number of genes in *V* which are mutated in sample *s*. This value would be equal to 1 if the genes in gene set *V* are all members of a perfect mutual exclusive pattern and |*V*| if all genes in *V* are mutated in all samples. The square root allows giving relatively higher mutual exclusivity scores to small subnetworks for which each gene is mutated in approximately the same number of samples.

**Figure 8:**
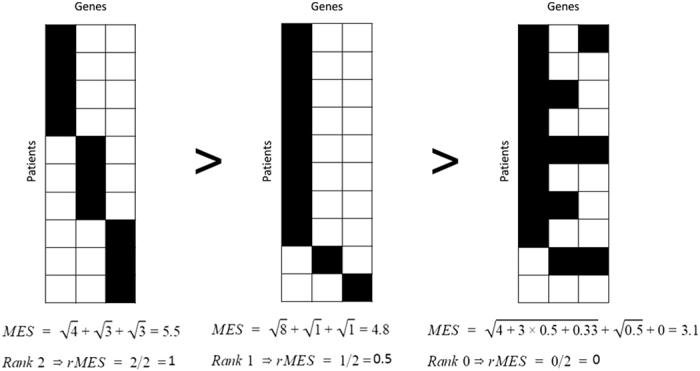
Calculation of *MES* and corresponding *rMES* scores for three different small subnetworks. Genes which make up the small subnetwork are represented as columns, patients are represented as rows. Genes with alterations in a specific patient are depicted as black tiles. Small subnetworks exhibiting perfect mutually exclusivity patterns (two most left small subnetworks) have higher *rMES* scores than small subnetworks with non-perfect mutual exclusivity patterns (most right small subnetwork). Also, small subnetworks having a more uniform distribution of gene alterations across patients have higher *rMES* scores as shown by the two most left small subnetworks.

Next, the *MES* are ranked from highest to lowest and their ranks are divided by the maximum rank (Fig. 8). We end up with a ranked *MES (rMES*) between zero and one where zero refers to the small subnetwork having the least evidence for mutual exclusivity and one refers to the small subnetwork having the most evidence for mutual exclusivity.

#### Reinforced learning

Using the *rMES* for each small subnetwork, the reinforced learning step updates gene scores based on two parameters: *reinforcement* and *forgetfulness*. The *reinforcement* is a parameter that determines the maximal value by which a gene score can be increased in the next iteration. The reinforcement is multiplied by the highest *rMES* score of all small subnetworks to which the gene belongs, so the gene score of genes which are consistently in small subnetworks with high *rMES* scores will further increase with iterations. The *forgetfulness* determines the fraction of the gene score that is retained in every subsequent iteration. This means that part of the gene score is effectively lost every iteration step and thus the gene scores of genes having persistently low scores will go to zero. To calculate gene scores, the following formula is used:


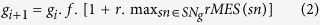


where *g*_*i*_ is the gene score at iteration *i*, *f* is the *forgetfulness*, *r* the *reinforcement*, *SN*_*g*_ the set of small subnetworks containing the gene. If the gene score resulting from the formula is larger than 1, it is topped off at 1 as the maximal gene score can never be larger than 1. The default parameters of the method are forgetfulness *f* = 0.995, reinforcement *r* = 0.005 and 5000 iterations. In general, the sum of forgetfulness and reinforcement should be close to 1 and the reinforcement should be small (smaller than 0.01). This because small values for forgetfulness or large values for reinforcement would make the algorithm prone to stochastic effects. Note that genes which are not part of any small subnetwork are assigned a value of zero for 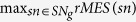.

In a final step we assign a rank to each gene that reflects the possibility of it being a driver gene. Hereto we exploit the fact that driver genes that exhibit mutual exclusivity tend to have a consistent increase in their gene score between iterations over time. Genes are ranked according to the maximal gene score they reach and in case of ties are based on how fast their score converges.

#### Bootstrapping

In order to eliminate predicted driver mutations which are likely artefacts of specific samples in the data, we perform a bootstrap analysis. Here, we randomly sample with replacement an equal number of tumor samples as in the original dataset and run SSA-ME on this new dataset. Each bootstrap dataset will contain some duplicate samples but will also lack some samples from the original dataset. For each dataset we generate and evaluate 1000 bootstrap datasets. We then evaluate these results by assessing at which minimal rank threshold (the rank threshold is the highest (worst) rank still considered in the calculation of the bootstrap support across all bootstrap results) a gene can attain a bootstrap support of 95% (selected in at least 95% of bootstrap results). We do this by gradually increasing the rank threshold. The final rank of the genes is based on the order in which this 95% bootstrap support is attained by the genes, the highest ranked gene being the gene which attained a bootstrap support of 95% using the most strict minimal rank threshold.

### Simulated data

To assess the performance of SSA-ME we used simulated data. The set of true positive driver genes was defined first by creating a target gene set of mutual exclusive genes which in biological terms corresponds to a driver pathway. The target gene set was generated using a random walker with restart (5% restart chance) to select genes from the local network neighborhood of a randomly selected gene until 20 interactions have been visited in a high quality human reference network. This high quality human reference network was composed of HINT^29^ version 3, Interactome (HI-II-14)^30^ and Reactome^31^ interaction data.

To mimic real tumor data, we counted the number of mutated genes present in each tumor sample in the TCGA 2012 study and assigned an equal number of alterations to random genes, thus conserving the distribution of mutated genes per sample. We added mutually exclusive mutations to genes present in the target gene set in 30% of the samples. Each sample had 5% chance to also be mutated in any of the other genes belonging to the mutually exclusivity gene set as we allowed for non-perfect mutual exclusivity module.

To evaluate the robustness of the method with respect to the used reference network, multiple simulated datasets were analyzed for different degrees of connectedness in the high quality human reference network: highly underconnected (50% of the edges were deleted from the reference network), mildly underconnected (25% of the edges deleted), lowly underconnected (10% edges deleted), original network (i.e. the high quality human reference network), lowly overconnected (10% additional random edges added to the reference network), mildly overconnected (25% additional edges) and highly overconnected (50% additional edges). We generated 100 different simulated datasets per network and ran SSA-ME. Performance was measured by receiver operating characteristic (ROC) curves.

To assess parameter sensitivity, we tested the effect of using different parameter combinations on the performance. This included 400 simulations for all combinations of reinforcement *r* (from 0.0005 to 0.0100 in steps of 0.0005) and forgetfulness *f* (from 0.99 to 0.9995 in steps of 0.0005). Performance for each parameter combination was measured using the area under the curve (AUC).

### TCGA Data

TCGA data was downloaded from GDAC Firehose^32^,^33^,^34^. We used somatic mutations annotated by MutatorAssesor^35^ and copy number alterations (CNAs) inferred with GISTIC^36^. We removed samples containing more than 500 genomic alterations to avoid taking into account hypermutator samples. In our analysis only copy number altered genes in samples with high-level thresholds (threshold 2 in GISTIC) for amplifications/deletions and for which copy number alteration showed a positive correlation (q < 0.05) with expression data were used. Prioritization results were obtained by running SSA-ME on a non-stringently filtered input set, consisting of all genes having at least one genetic alteration (mutation or amplification/deletion) in the dataset. As a high quality human reference network we compiled information data from HINT^29^ version 3, Interactome (HI-II-14)^30^ and Reactome^31^. Results for MutSigCV and MutSig2CV were downloaded from GDAC Firehose^37^,^38^. Results for Mutex were taken from supplementary of the original paper^20^. Results for Oncodrive-FM were obtained by running Oncodrive-FM using default settings and functional impact scores (SIFT^39^, mutation assessor^35^ and PolyPhen2^40^).

#### Patterns of mutual exclusivity

SSA-ME searches for small subnetworks that display a high degree of mutual exclusivity. To visualize the patterns of mutual exclusivity for any prioritized gene, SSA-ME selects the five best subnetworks (with highest MES score) to which that prioritized gene belongs. In many cases the five best small subnetworks to which the prioritized gene belongs, overlap and thus the union of these genes is used as a pattern of mutual exclusivity with the prioritized gene. However, as we do not explicitly impose the constraint that within such a union there should be mutual exclusivity, there is no guarantee that all genes within the retrieved pattern are mutually exclusive. It is perfectly possible that such a union consist of two separate patterns of mutual exclusivity, each involving the prioritized gene.

### Data Availability

SSA-ME software is available at https://github.com/spulido99/SSA. CADD scores version 1.3 were downloaded from http://cadd.gs.washington.edu/. The TCGA Pan-Cancer datasets are publicly available at https://gdac.broadinstitute.org/.

## Additional Information

**How to cite this article**: Pulido-Tamayo, S. *et al.* SSA-ME Detection of cancer driver genes using mutual exclusivity by small subnetwork analysis. *Sci. Rep.* **6**, 36257; doi: 10.1038/srep36257 (2016).

**Publisher’s note:** Springer Nature remains neutral with regard to jurisdictional claims in published maps and institutional affiliations.

## Supplementary Material

Supplementary Information
