# Supplementary material for: SSA-ME Detection of cancer driver genes using mutual exclusivity by small subnetwork analysis
Source: Sci Rep. 2016 Nov 3;6:36257. doi: 10.1038/srep36257 (PMC5093737; doi:10.1038/srep36257)
Supplement: Supplementary Information [file srep36257-s1.pdf]

SSA.ME Detection of cancer mutual exclusivity patterns by small subnetwork analysis

Sergio Pulido-Tamayo[1,2,3,5,6,†], Bram Weytjens[1,2,3,5 †], Dries De Maeyer[1,2,3,5], Kathleen Marchal[1,2,3,4*]

[1] Dept. of Information Technology (INTEC, iMINDS), UGent, Ghent, 9052, Belgium

[2] Dept. of Plant Biotechnology and Bioinformatics, Ghent University, Technologiepark 927, 9052 Gent, Belgium

[3] Bioinformatics Institute Ghent, Technologiepark 927, 9052 Ghent, Belgium

[4] Department of Genetics, University of Pretoria, Hatfield Campus, Pretoria 0028, South Africa

[5] Dept. of Microbial and Molecular Systems, KU Leuven, Kasteelpark Arenberg 20, B-3001 Leuven, Belgium

[6] Grupo de Investigación en Ciencias Biológicas y Bioprocesos (Cibiop), Universidad EAFIT, Carrera 49 N° 7 Sur-50, Medellín, Colombia

[*] Corresponding author: Kathleen Marchal. E-mail: kathleen.marchal@intec.ugent.be

[†] These authors contributed equally to this work.

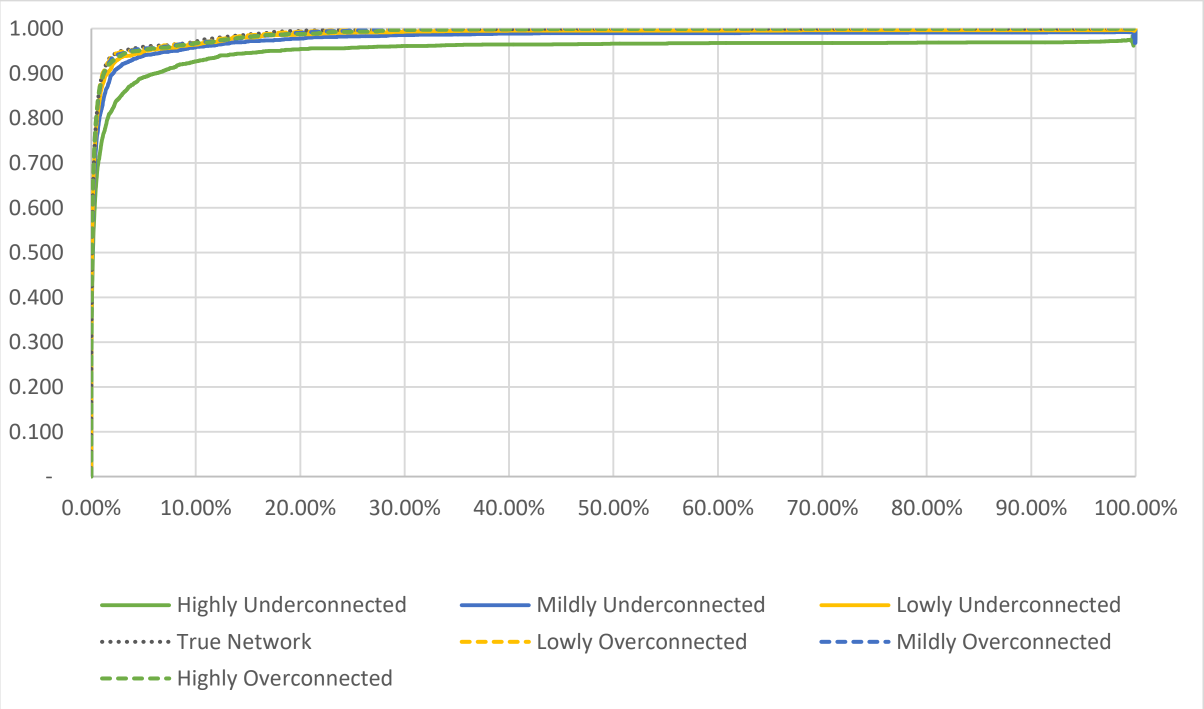

Supplementary Figure 1. Robustness of the predictions with respect to the used reference network. The X-axis represents 1-specificity and the Y-axis represents sensitivity. Underconnected networks result in a lower performance while overconnected networks result in similar, although lower, performance to the true network. Complete ROC curve.

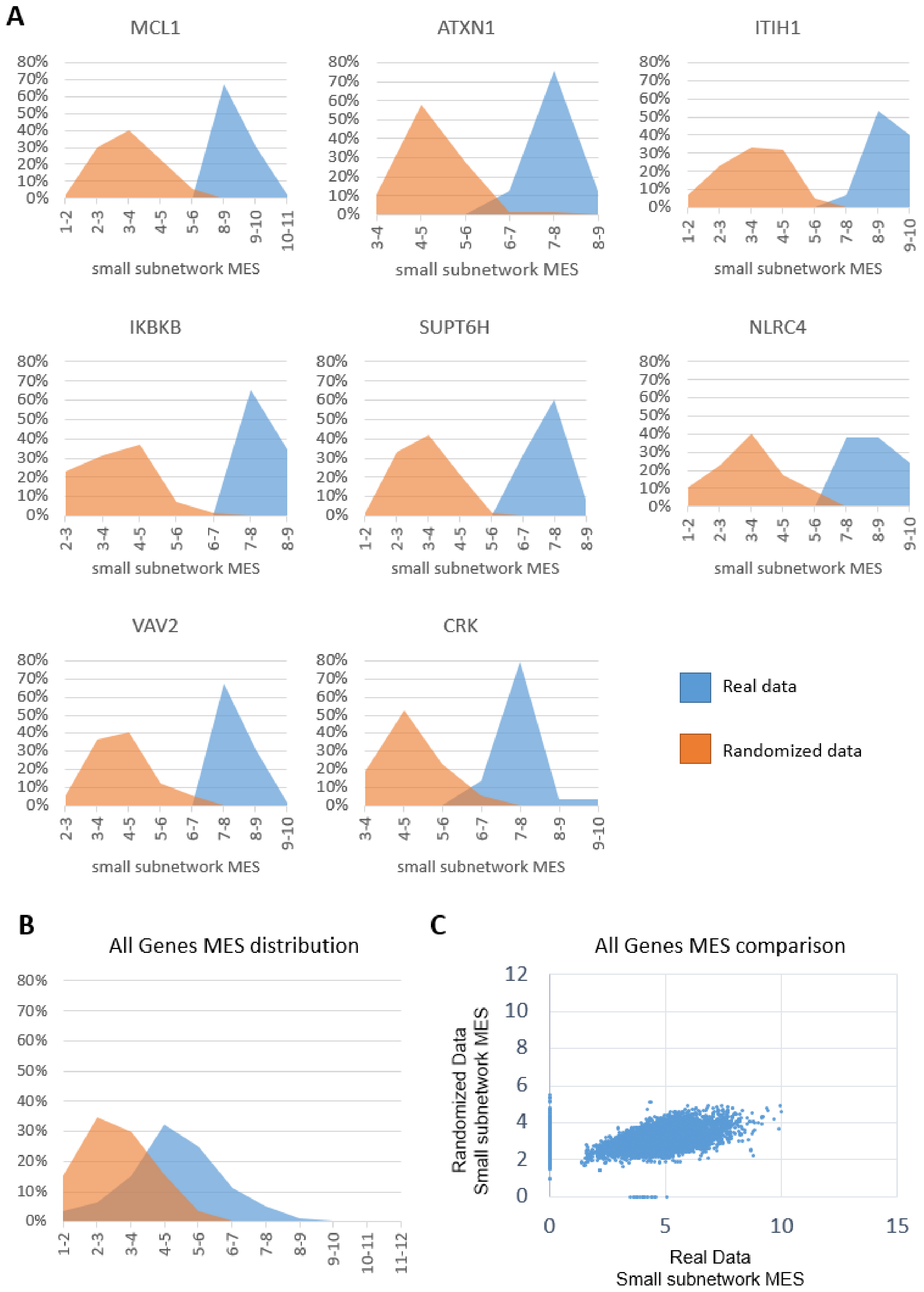

Supplementary Figure 2. Differences in mutual exclusivity scores of small subnetworks derived from respectively real and randomized datasets. Note that in order to have complete information about the mutual exclusivity scores of the small subnetworks to which a specific gene can be assigned we, for each randomized dataset and also for the real data, ran the algorithm 100 times and each time retained the mutual exclusivity score of the subnetwork with which that gene is associated upon convergence of the method. This leads to the score distributions depicted

in these figures. A) Distributions of mutual exclusivity scores of the small subnetworks in randomized datasets (orange) and the real dataset (blue) for some putative driver mutations prioritized by SSA-ME. B) Distribution of mutual exclusivity scores of the small subnetworks in randomized datasets (orange) and the real dataset (blue) for all genes. C) Graph showing per gene the average score of the small subnetworks it belonged to upon convergence of the algorithm as derived from the real data (X-axis) and from the randomized data (Y-axis). Mutual exclusivity scores are normalized by the size of the small subnetworks. Randomization were performed by shuffling gene names. See supplementary table 2 for the confidence intervals of the differences in mutual exclusivity scores between randomized datasets and the real dataset for the putative driver genes and all genes together.

Pan-cancer datasets

Apart from the BRCA dataset, we also analyzed data from the BLCA, COADREAD, GBM, HNSC, KIRC, LAML, LUAD, LUSC, OV, UCEC and STAD datasets. All datasets used for these analyses were downloaded from GDAC firehose. Resulting networks, prioritized genes and PPV value plots can be found in supplementary figures 3-13 and supplementary tables 3-13.

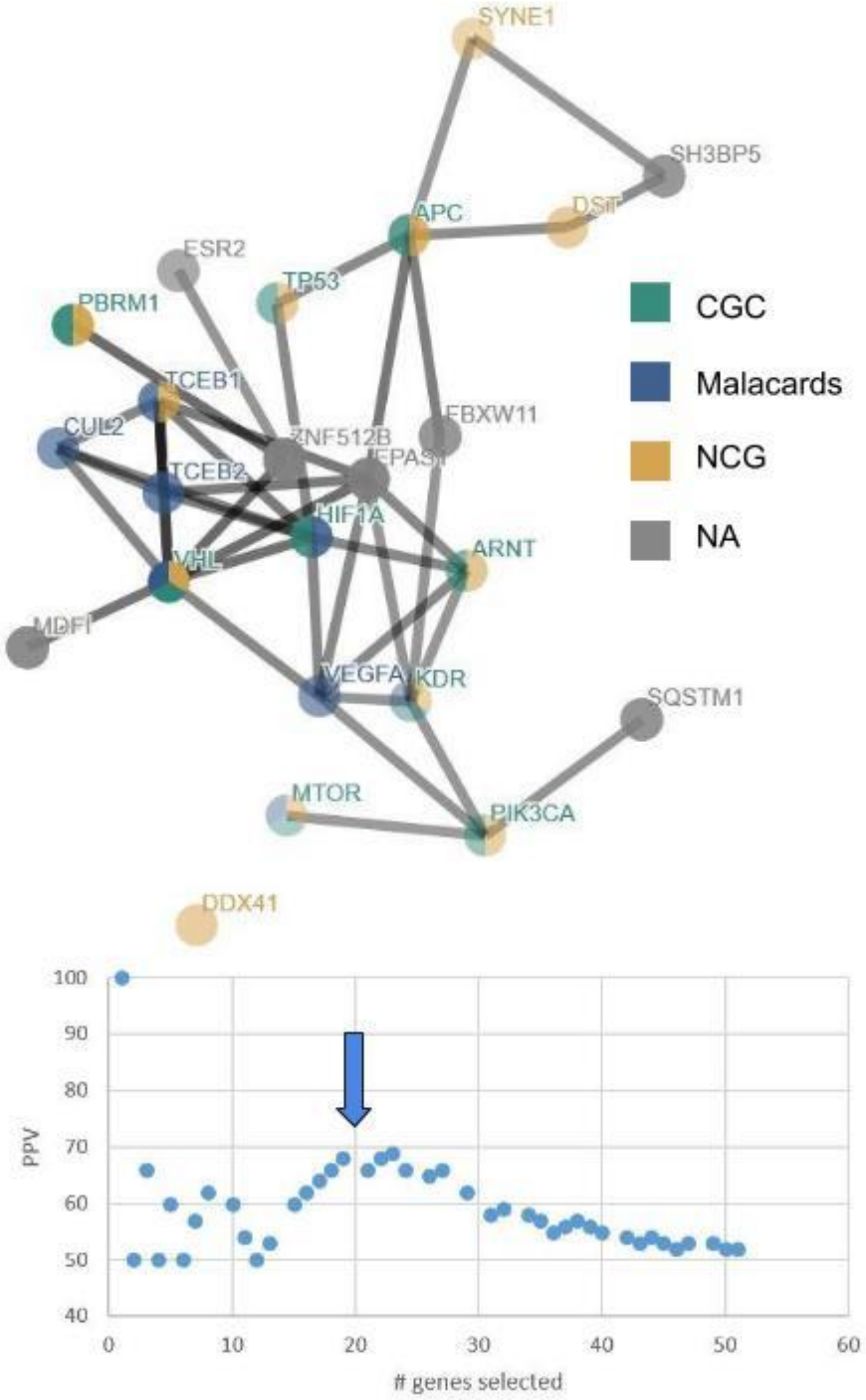

Supplementary Figure 3. Network of selected genes and PPV plot for kidney renal clear cell carcinoma (KIRC)

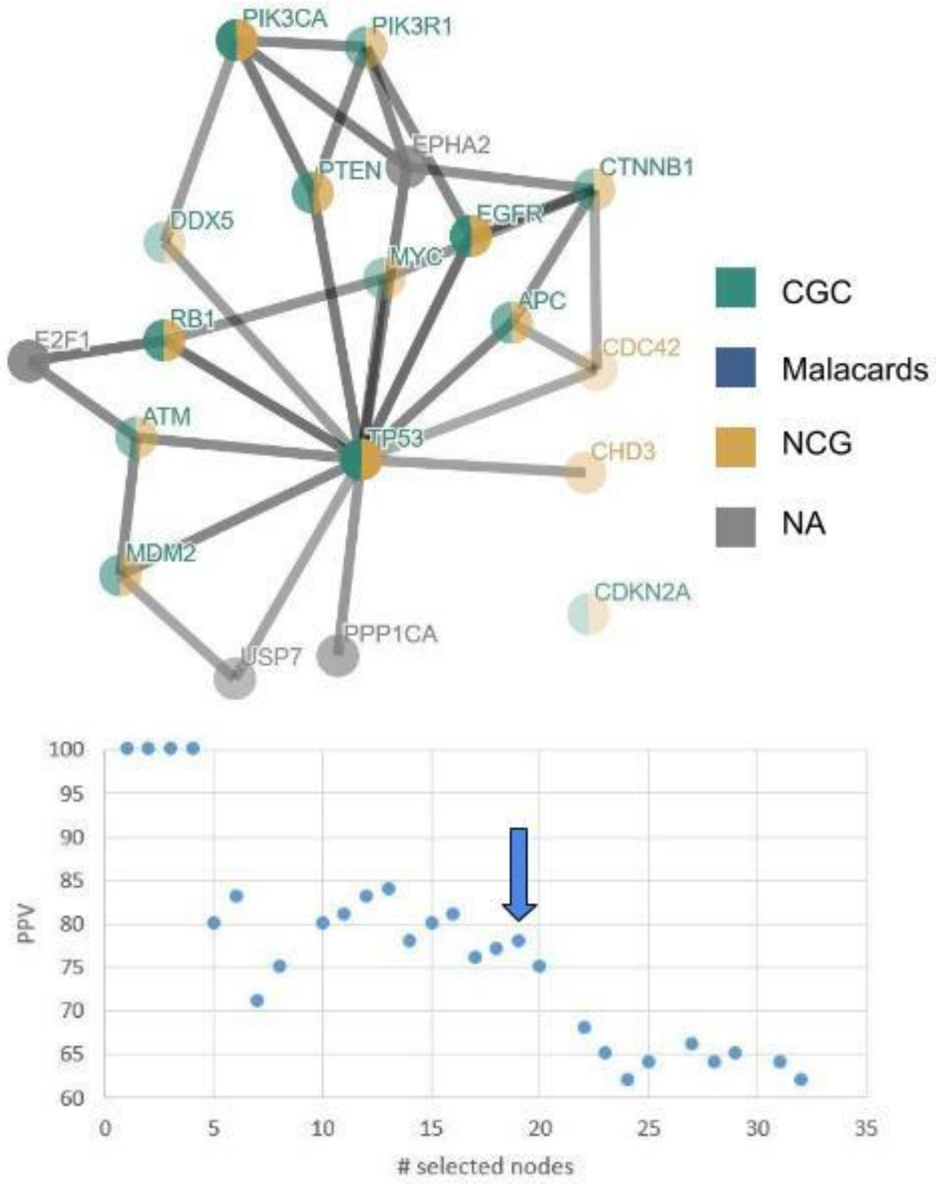

Supplementary Figure 4. Network of selected genes and PPV plot for head and Neck squamous cell carcinoma (HNSC)

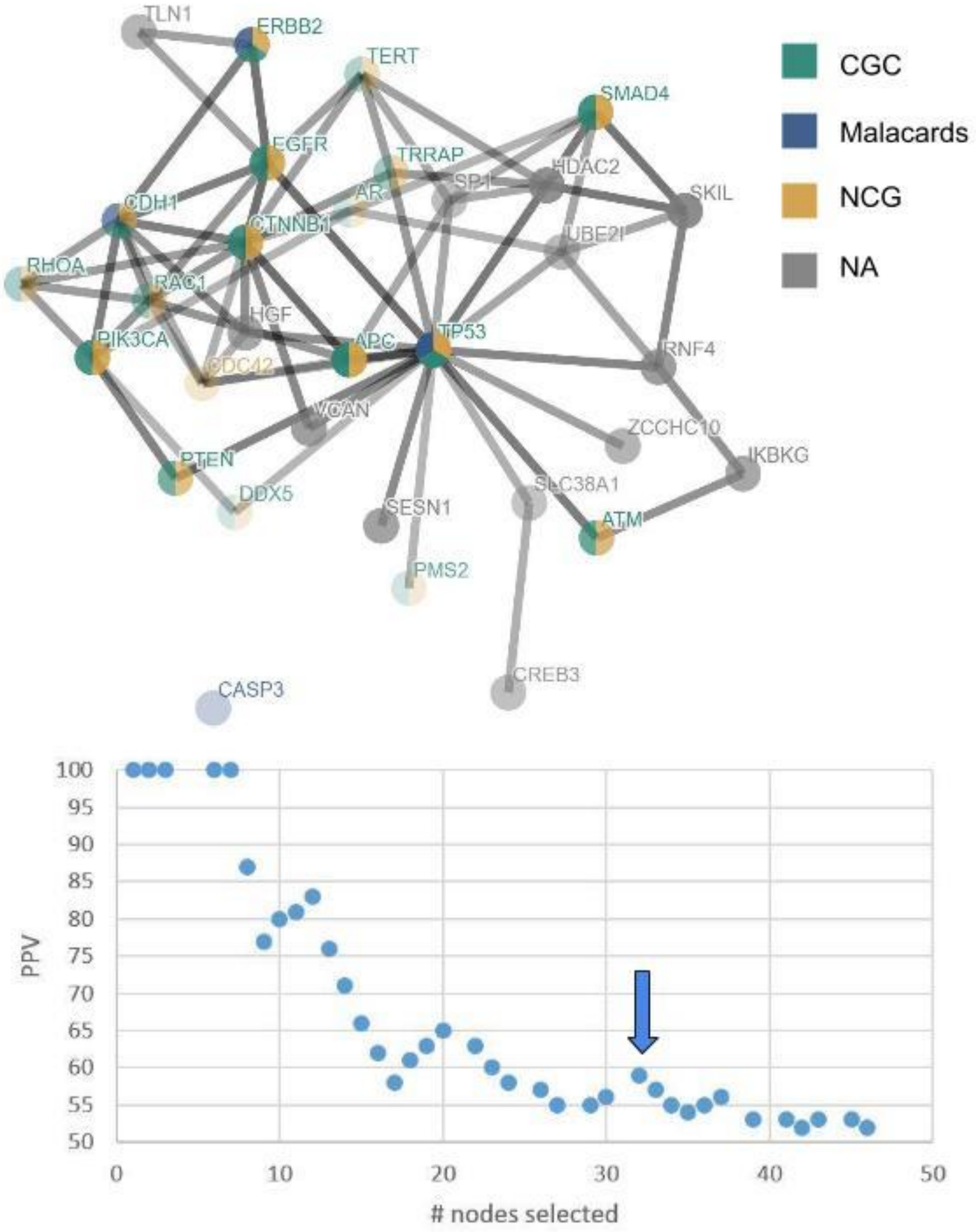

Supplementary Figure 5. Network of selected genes and PPV plot for stomach adenocarcinoma (STAD)

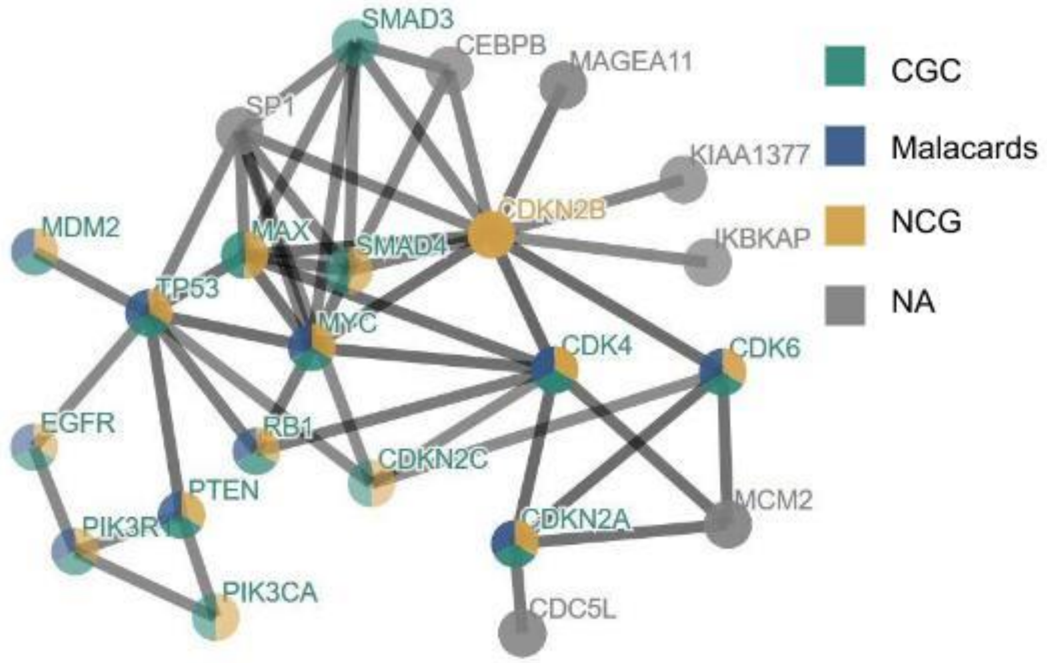

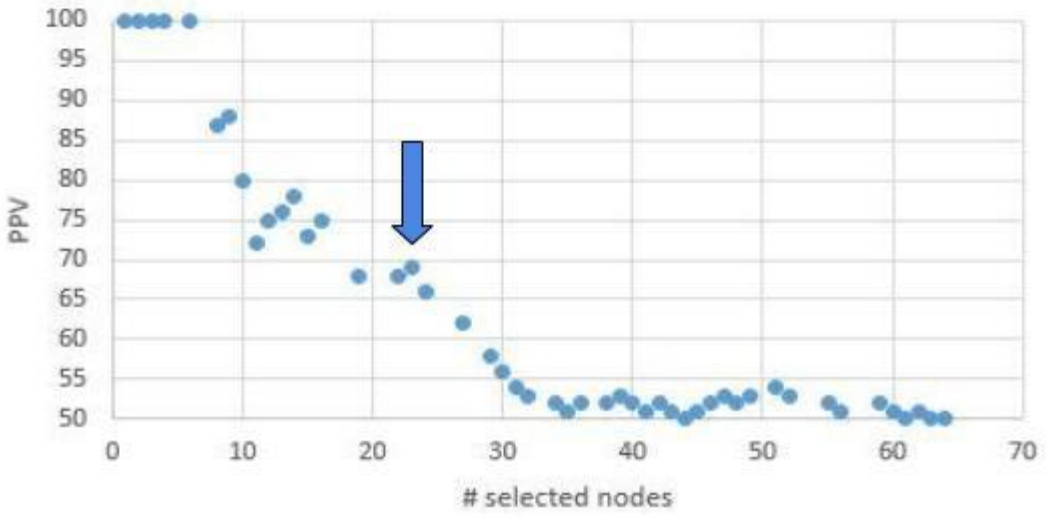

Supplementary Figure 6. Network of selected genes and PPV plot for glioblastoma multiforme (GBM)

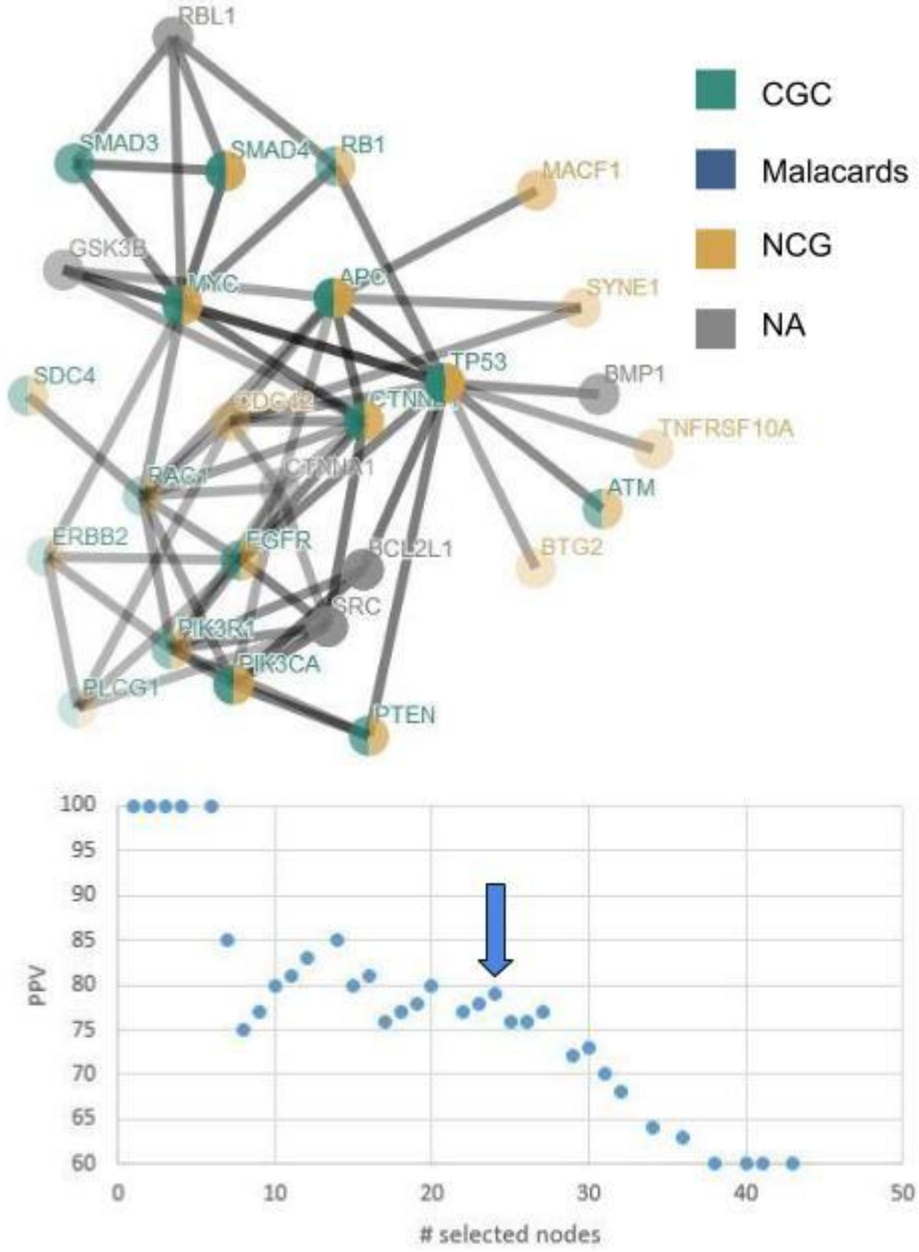

Supplementary Figure 7. Network of selected genes and PPV plot for colorectal adenocarcinoma (COADREAD)

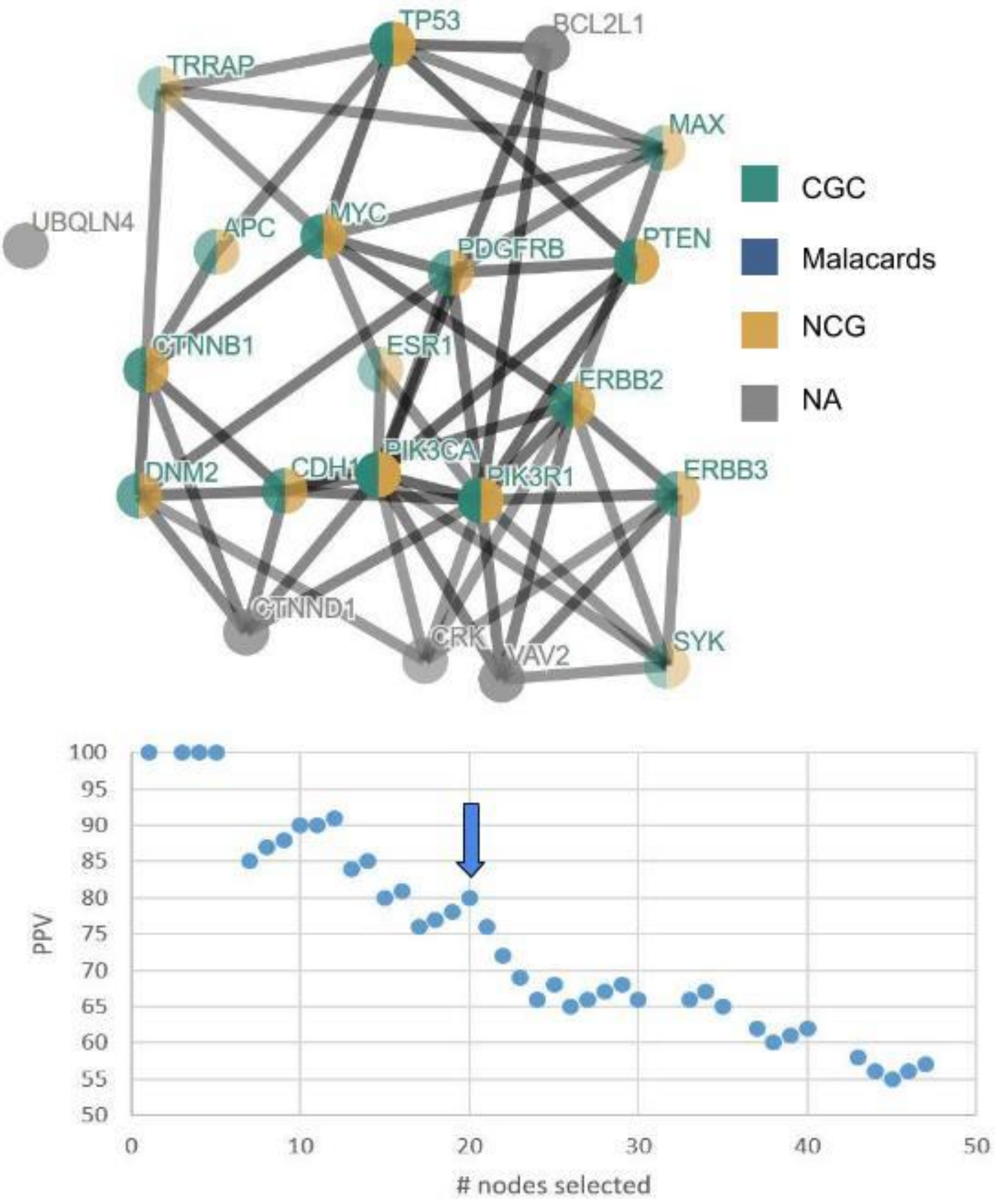

Supplementary Figure 8. Network of selected genes and PPV plot for uterine Corpus Endometrial Carcinoma (UCEC)

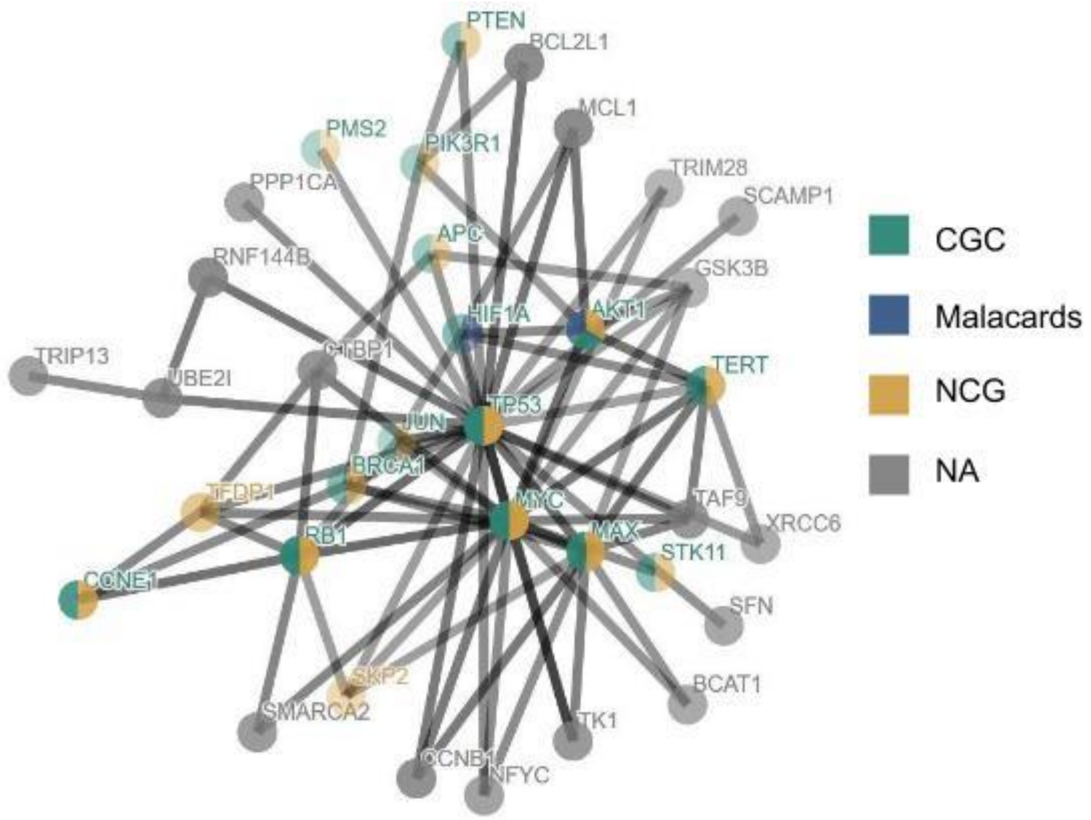

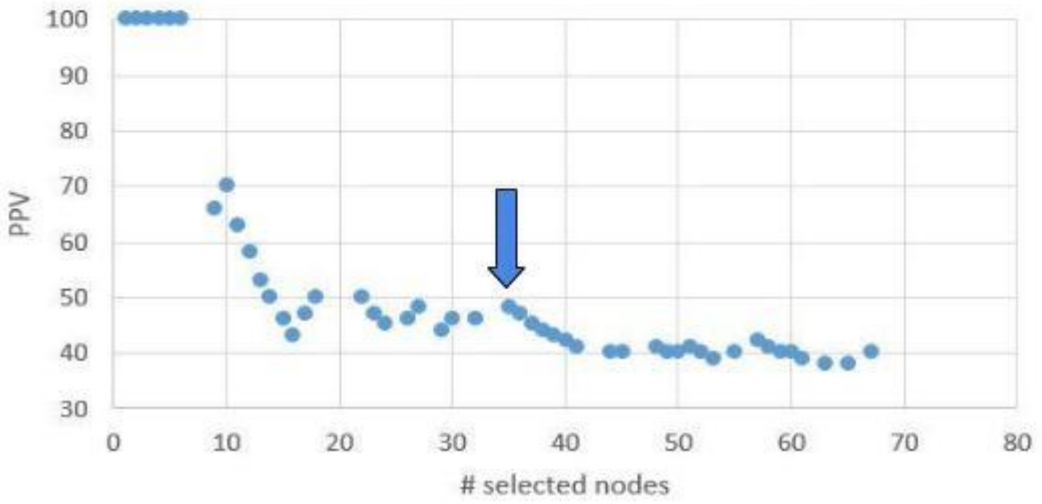

Supplementary Figure 9. Network of selected genes and PPV plot for ovarian serous cystadenocarcinoma (OV)

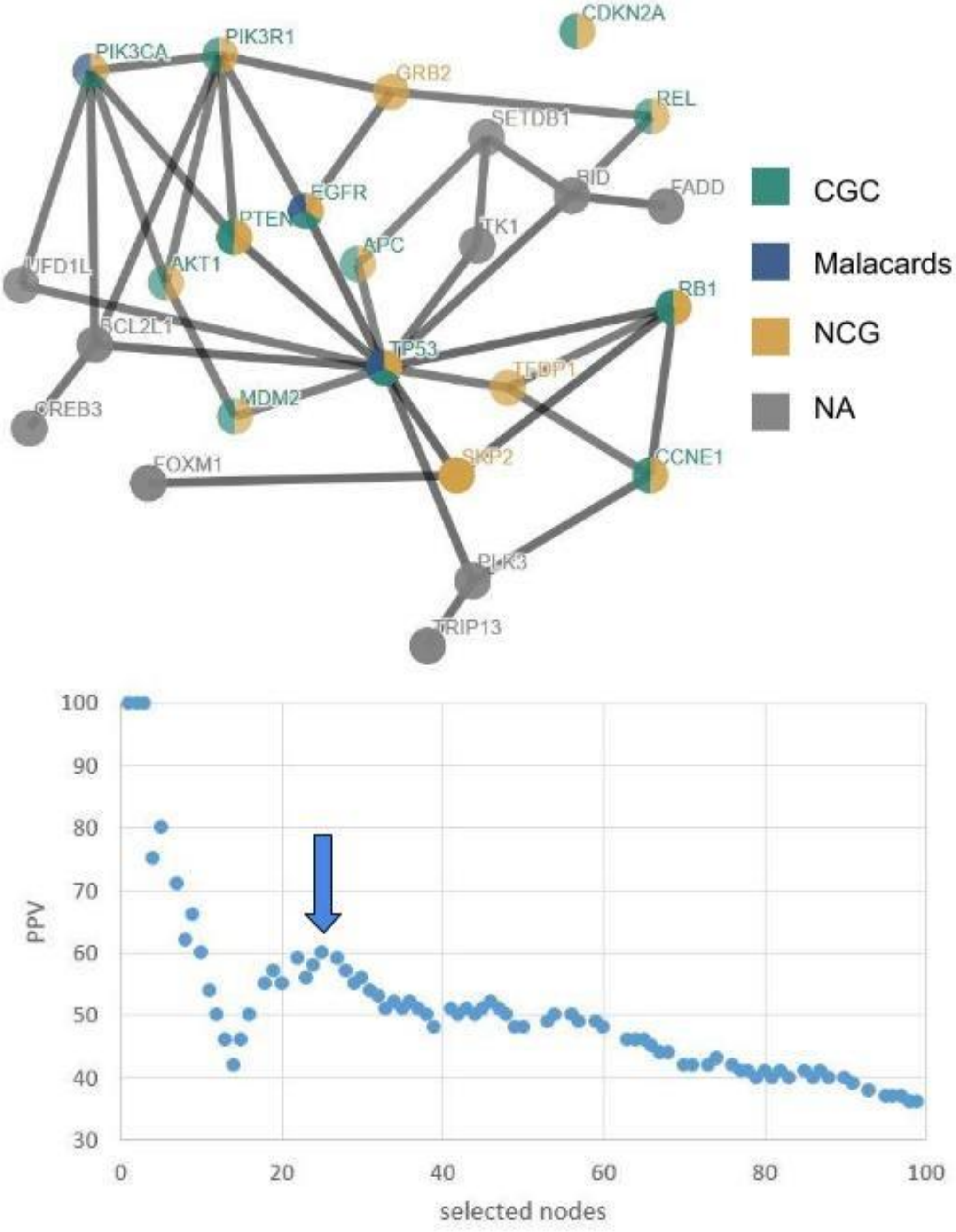

Supplementary Figure 10. Network of selected genes and PPV plot for lung squamous cell carcinoma (LUSC)

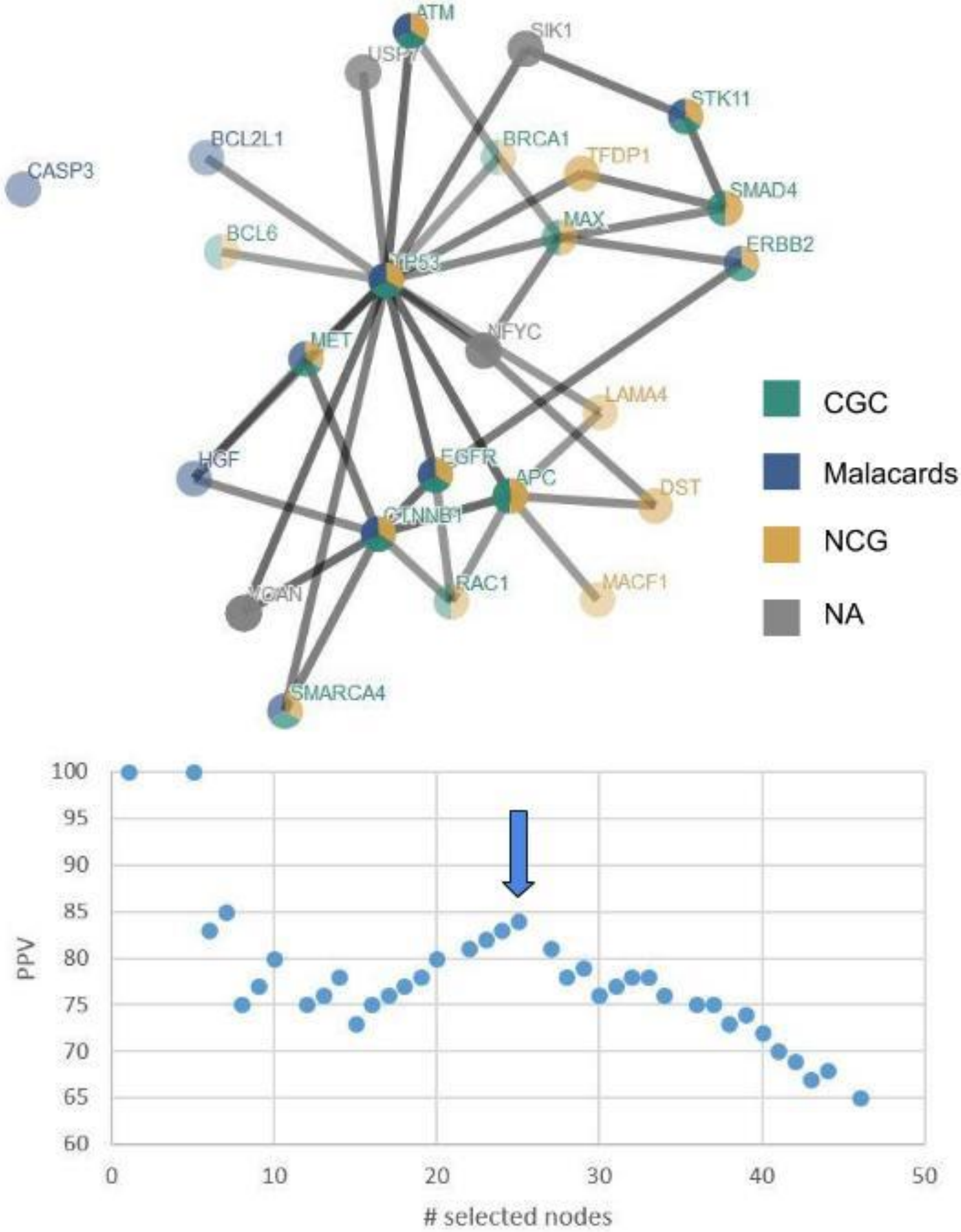

Supplementary Figure 11. Network of selected genes and PPV plot for lung adenocarcinoma (LUAD)

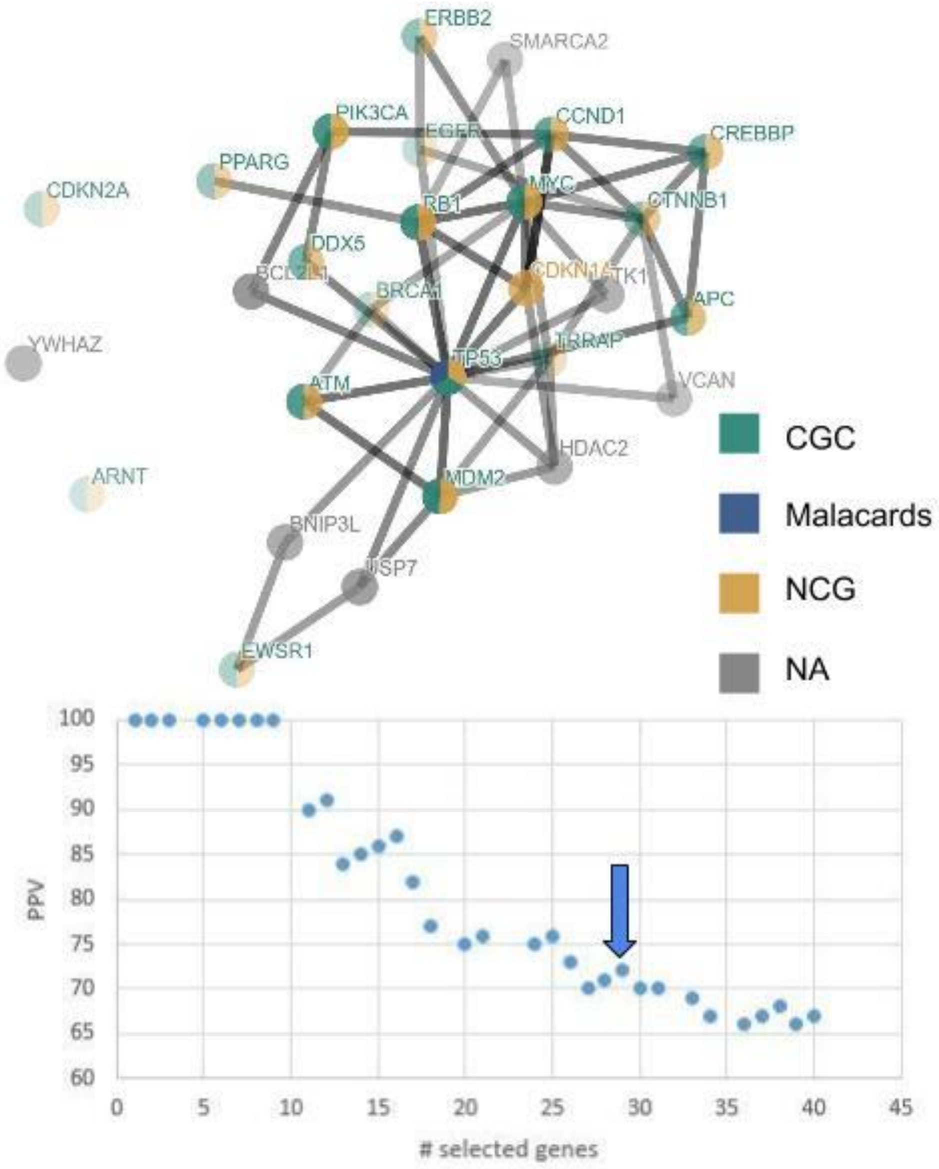

Supplementary Figure 12. Network of selected genes and PPV plot for bladder urothelial carcinoma (BLCA)

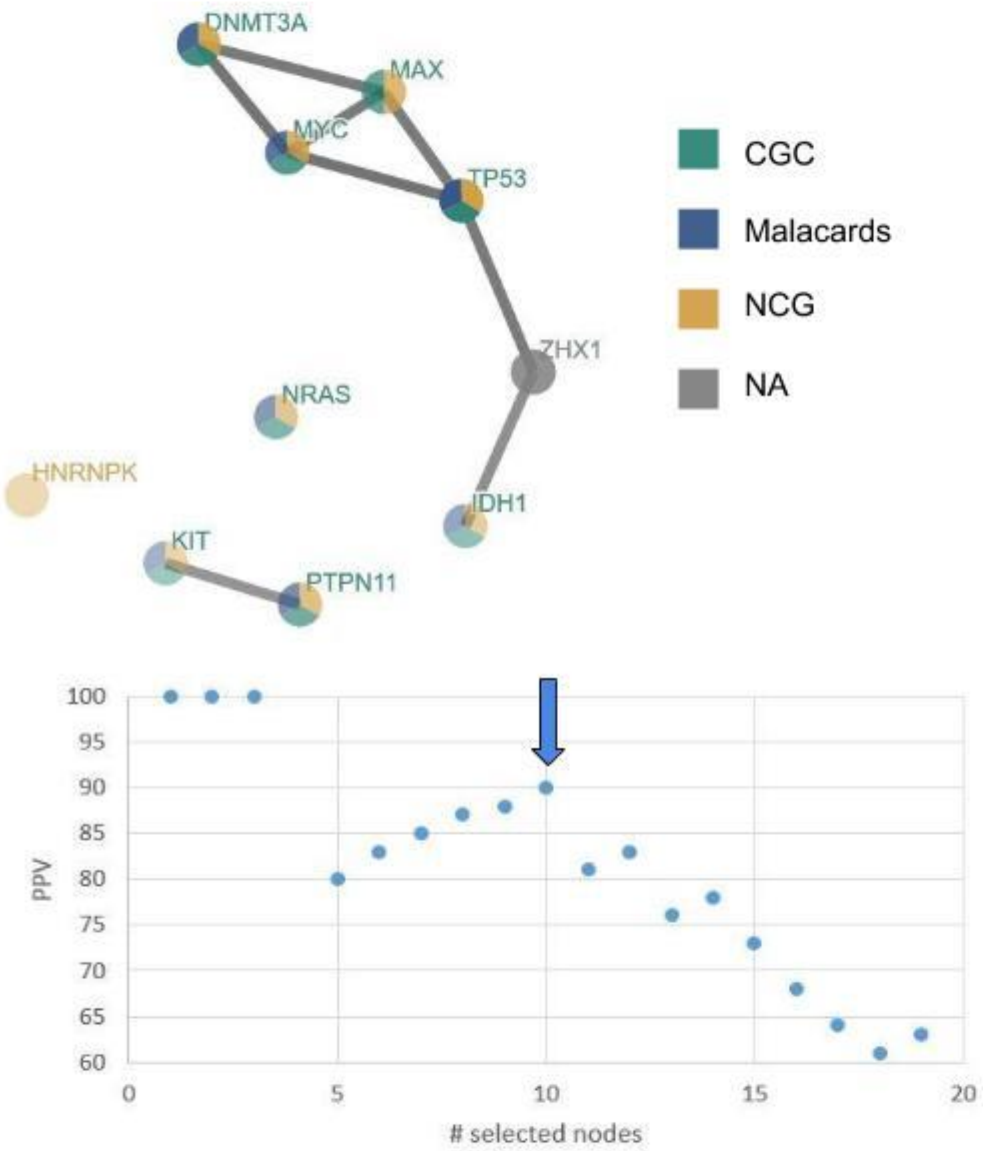

Supplementary Figure 13. Network of selected genes and PPV plot for acute myeloid leukemia (LAML)

Literature-based evidence for predicted cancer drivers in the breast cancer dataset

Of the 34 ranked genes, 8 genes were not listed in cancer gene databases (*MCL1*, *GAB2*, RPS*6KB1*, *CRK*, *NGFR*, *EPHA2*, *VAV2* and *UFD1L*) based on CGC version 77, NCG 5.0 or the Malacards Breast Cancer category version 1.11.724. These genes are discussed below. Some of these are well known cancer drivers not reported in CGC, because they contain CNVs rather than somatic mutations. For selected genes which are not listed in cancer gene databases, for which the mutations are mainly SNP's and which have at least 20 SNP's in all pan-cancer datasets combined (to ensure the pattern can be visually convincing), we show the uncovered mutual exclusivity profiles (*EPHA2* and *VAV2* showed).

MCL1 was found frequently (64 times) amplified in the dataset. *MCL1* is involved in apoptosis modulation and signaling[1]. Its alterations by CNVs have been reported in literature before[2]. It has been associated with a number of cancers because of its involvement in the regulation of apoptosis versus cell survival[3].

Both ***GAB2*** and *PAK1* were frequently amplified (respectively 58 and 61 times) in the TCGA breast cancer dataset. Both genes belong to the same amplicon as the well-known breast cancer driver *CCND1*[4], which was in concordance also frequently amplified. However, because it cannot be excluded that more genes in the same amplicon are causal to cancer and because *CCND1*, *GAB2*, and *PAK1* each show a strong mutual exclusivity with a subset of selected genes closely related in the network, each of them might act independently from one another as a true driver. Whereas both *CCND1*, a regulatory protein involved in mitosis, and *PAK1*, a protein belonging to the family of serine/threonine p21-activating kinases that are involved in cytoskeleton reorganization and nuclear signaling, have been reported in at least one of the cancer related databases, *GAB2* is not. *GAB2* was prioritized because of its mutual exclusivity and close network connectivity with amongst others *PIK3CA*, *PTEN* and *EPHA2* (Fig. 4). *GAB2* is a scaffolding adapter protein that transduces cellular signals between receptors (tyrosine kinase receptors) and intracellular downstream effectors (*PI3K*, *SpH2*) and is required for efficient *ErbB2*-driven mammary tumorigenesis and metastatic spread by acting downstream of *ErbB2*[5,6]. Interestingly, it was also shown that a focal amplification of *GAB2* independently of *CCND1* in breast tumors contributes to diverse oncogenic phenotypes in breast cancer by activating, amongst others, the PI3K pathway, further confirming the role of *GAB2* as primary driver in breast cancer[7].

RPS6KB1 was found to be frequently (77 times) amplified in the TCGA breast cancer dataset. *RPS6KB1*, encoding a ribosomal S6 kinase 1 (S6K1) is a member of the frequently mutated PI3K pathway and has been reported to be involved in cell proliferation and protein translation. A link between the S6K1 function and cancer was suggested by the finding that *RPS6KB1* resided in the chromosomal region 17q22-17q23 and was often amplified in lung and breast cancers[8,9].

Other genes we prioritized were not listed in cancer gene databases but were previously associated with cancer because of their expression behavior expression.

The signaling adaptor protein **Crk** has been shown to play an important role in various human cancers. In the used breast cancer dataset *CRK* only had one SNP. The CRK family proteins all act as molecular bridges between tyrosine kinases and their substrates and modulate the specificity and stoichiometry of signaling processes. Evidence suggests that cellular Crk proteins are overexpressed in human tumors and that expression levels correlate with aggressive and malignant behavior of cancer cells[10]. Using RNAi-mediated knockdown, Fathers et al.[11] have shown in their study that *CRK* is required for cell migration and invasion of metastatic breast cancer cells *in vitro* and for metastatic growth *in vivo*. However, a mechanistic understanding of Crk proteins in cancer progression *in vivo* is still lacking, partly because of the highly pleiotropic nature of Crk signaling[12].

NGFR (nerve growth factor receptor). It had 1 SNP in the BRCA dataset. *NGFR* inactivates p53 by promoting its MDM2-mediated ubiquitin dependent proteolysis and by directly binding to its central DNA binding domain and preventing DNA-binding activity. Biologically, cancer

cells hijack the negative feedback regulation of p53 by *NGFR* to their growth advantage, as down regulation of *NGFR* induces p53-dependent apoptosis and cell growth arrest as well as suppressed tumor growth[13]. Overexpression of *NGFR* has been observed in many metastatic cancers and promotes tumor migration and invasion[14-16].

The **EphA2** receptor is involved in multiple cross-talks with other cellular networks including EGFR, FAK and VEGF pathways, with which it collaborates to stimulate cell migration, invasion and metastasis[17]. It had 7 mutations in the BRCA dataset (3 SNP's, 1 amplification and 3 deletions). While its overexpression has been correlated to stem-like properties of cells and tumor malignancy as for instance in colon cancer, less information is available on the role of *EPHA2* as a driver gene. We did prioritize *EPHA2* as a driver in breast cancer, despite its relatively low number of mutations. This because it showed (near) perfect mutual exclusivity with, amongst others, the well-known drivers *PIK3CA*, *PTEN*, *GAB2* and *RP6KB1*, and all members of the PI3K pathway known to act downstream of *EPHA2*. These results were confirmed by the pan-cancer datasets from which it appears that *EPHA2* was mainly highly mutated in HNSC (Head and neck squamous cell carcinoma) (Fig. 4). A recent study shows that rare SNPs in receptor tyrosine kinases, amongst which *EPHA2*, can be associated with negative outcome. This further points towards the clinical relevance of these less frequently mutated drivers[18]. See Fig. 4 in the main text for the retrieved mutual exclusivity pattern of *EPHA2* in BRCA and in all pan-cancer datasets.

VAV2 was also prioritized in the breast cancer dataset but rarely mutated (only 2 mutations in BRCA). *VAV2* is a gene involved in altering cell shape and migration and has previously been associated with metastasis in breast cancer[19]. It was prioritized because of its association with *PIK3CA* and *ERBB2*, a signaling subnetwork that was shown in literature to be involved in ovarian tumor cell migration and growth through activation of *PI3K* in HER2 ovarian tumors. This activation leads to the recruitment of actin and actinin to ERBB2, which then colocalizes with the VAV2 guanine nucleotide exchange factor to induce Rac1 and Ras signaling and the concomitant activation of ovarian tumor cell migration and growth[20]. See Supplementary Fig. 15 for the retrieved mutual exclusivity pattern of *VAV2*.

UFD1L was prioritized in BRCA but has only 1 SNP. As there is only limited evidence to support the involvement of *UFD1L* in tumorigenesis[21] we cannot rule out *UFD1L* is a false positive.

Literature-based evidence for frequently predicted cancer drivers in the pan-cancer dataset

Here we only focus on a detailed description of genes that were prioritized recurrently in the different pan-cancer datasets and that were not yet mentioned as drivers in the used cancer gene reference databases. We use the same criterion for showing mutual exclusivity patterns as in the previous paragraph (*VCAN* showed).

Versican (***VCAN***) was selected in three out of twelve different pan-cancer datasets (LUAD STAD, BLCA) and fell just below the PPV cutoff value in UCEC. *VCAN* is a major component of the extracellular matrix (EM) involved in cell adhesion, proliferation, migration and angiogenesis. Increased *VCAN* expression has been observed in a wide range of malignant

tumors and has been associated with both cancer relapse and poor patient outcomes[22-24]. Despite its well documented role in triggering tumor proliferation[25,26], *VCAN* itself is not frequently mutated (mutations in 215 samples on a total of 5986 samples in the used pan-cancer datasets). In the LUAD dataset, *VCAN* was interacting and mutual exclusive with *EGFR, BCL9* and *CTNNB1* (b1 catenin). CTNNB1 is a well-known driver gene that plays a central molecule in the wnt pathway and that is involved in the transcriptional regulation of *VCAN*[27]. The uncovered mutual exclusivity pattern can be seen in Supplementary Fig. 15.

***BCL2L1* (BCL2 like 1, BCLX, BCLXL),** belongs together with Mcl-1 to the Bcl2 family. *BCL2L1* is an anti-apoptotic gene that has just like *MCL1* has been observed to be amplified in a variety of cancers. This is in accordance with our findings where *BCL2L1* was selected as a potential driver gene in 66% of the pan-cancer datasets (OV, BLCA, COADREAD, LUAD, UCEC and LUSC) in which it mostly had gains of copy number. Overexpression of anti-apoptotic Bcl-2 proteins in cancers tilts the apoptosis signaling pathway towards cell survival. *BCL2L1* is, next to its role in promoting cancer cell survival by suppressing apoptosis, also involved in promoting metastasis in a way that is independent of the anti-apoptotic activity[2].

UBE2I Was prioritized in the ovarium (OV) and in the stomach adenocarcinoma (STAD) pan-cancer datasets (as a linker gene). The ubiquitin-conjugating enzyme 9 (Ubc9), the sole conjugating enzyme for sumoylation, regulates protein function and plays an important role in sumoylation-mediated cellular pathways. Sumoylation plays a key role in DNA repair and tumorigenesis. Indeed, overexpressing Ubc9 has been shown to contribute to EOC progression and cell proliferation through the PI3K/Akt pathway[28]. In addition, the SUMO pathway mediated by Ubc9 was shown to critically contribute to the transformed phenotype of KRAS mutant cells[29]. *UBE2I* was prioritized in OV because of its association with *TP53* and *RNF144B*. The latter protein is an E3 ubiquitin-protein ligase that accepts ubiquitin from the E2 ubiquitin-conjugating enzymes UBE2L3 and UBE2L6 and then directly transfers the ubiquitin to targeted substrates, thereby promoting their degradation. It induces apoptosis via a p53/TP53-dependent mechanism and affects cell death by affecting the ubiquitin-dependent stability of BAX, a pro-apoptotic protein[30].

Comparison with MEMo

In order to compare the results of SSA-ME with those of MEMo, a method that searches for mutual exclusivity patterns using an interaction network, we obtained the results from MEMo on the TCGA 2012 breast cancer dataset[19] and ran SSA-ME on the same TCGA 2012 dataset, which was obtained directly from the TCGA breast cancer analysis portal. To maximize comparability between our results and those of MEMo, we reproduced to the best possible extent the filtering approach and network of the original MEMo study to run SSA-ME.

The used network is a non-curated network consisting of Reactome[31], Panther[32], KEGG[33], INOH[34] and interactions from non-curated sources (like high-throughput derived protein–protein interactions, gene co-expression, protein domain interaction, GO annotations, and text-mined protein interactions)[35]. The genetic alteration data was prepared according to the description in the original paper, i.e. only retaining genes that were altered in at least ten samples.

Just like Mutex, MEMo is primarily designed to detect patterns of mutual exclusivity but does not explicitly extract drivers. To compare the results of MEMo with these of SSA-ME and because of the high similarity of the mutual exclusivity patterns detected by MEMo in the original paper (patterns consisting of maximally 8 genes that varied in most cases in no more than one gene), we collapsed the 23 genes of all patterns found by MEMo and depicted them as a network (Supplementary Fig. 14A). The subnetwork obtained by SSA-ME consisted of 33 genes (applying a FDR cutoff, as described in the main text) of which 18 were also found in the MEMo network (Supplementary Fig. 14B). 5 genes retrieved by MEMo were not detected by SSA-ME: 3 genes (*NBN*, *CHECK2* and *MDM4)* because they were no longer present in the filtered list we used as input, whereas they must have been present in the original input of MEMo: in contrast to what has been described in the original TGCA paper we found these genes to be mutated in less than 2 samples and therefore removed them from our analysis, the score of *ATM* just fell below the chosen threshold of the ranked list of SSA-ME (*ATM* ranked 36 whereas with the chosen cut-off we only retained the 33 top ranked genes) and *ATK3* was truly missed in our analysis as the small subnetworks to which it belonged never received consistently high scores during subsequent iteration steps.

On the other hand, we found 10 additional genes that were not retrieved by MEMo. Some of these additional genes had previously been associated to breast cancer (*AR* and *ESR1)* or to cancer in general (*MUC4* and *CCDN1)*. The reason why we detect more genes than MEMo is partially due to the choice of the cut-off, but also because of the inherent differences in selection criteria between the methods: MEMo searches for patterns of mutual exclusivity in which all genes need to be mutually exclusive which each other (have to pass a permutation test) whereas the mutual exclusivity criteria of SSA-ME are less stringent. Also, our method does not require stringent filtering which leaves the possibility of selecting rarely mutated genes.

These results thus show that SSA-ME is able to reproduce largely the same results as MEMo, provided the same input data is used. Genes that are highly ranked by MEMo are also highly ranked by SSA-ME.

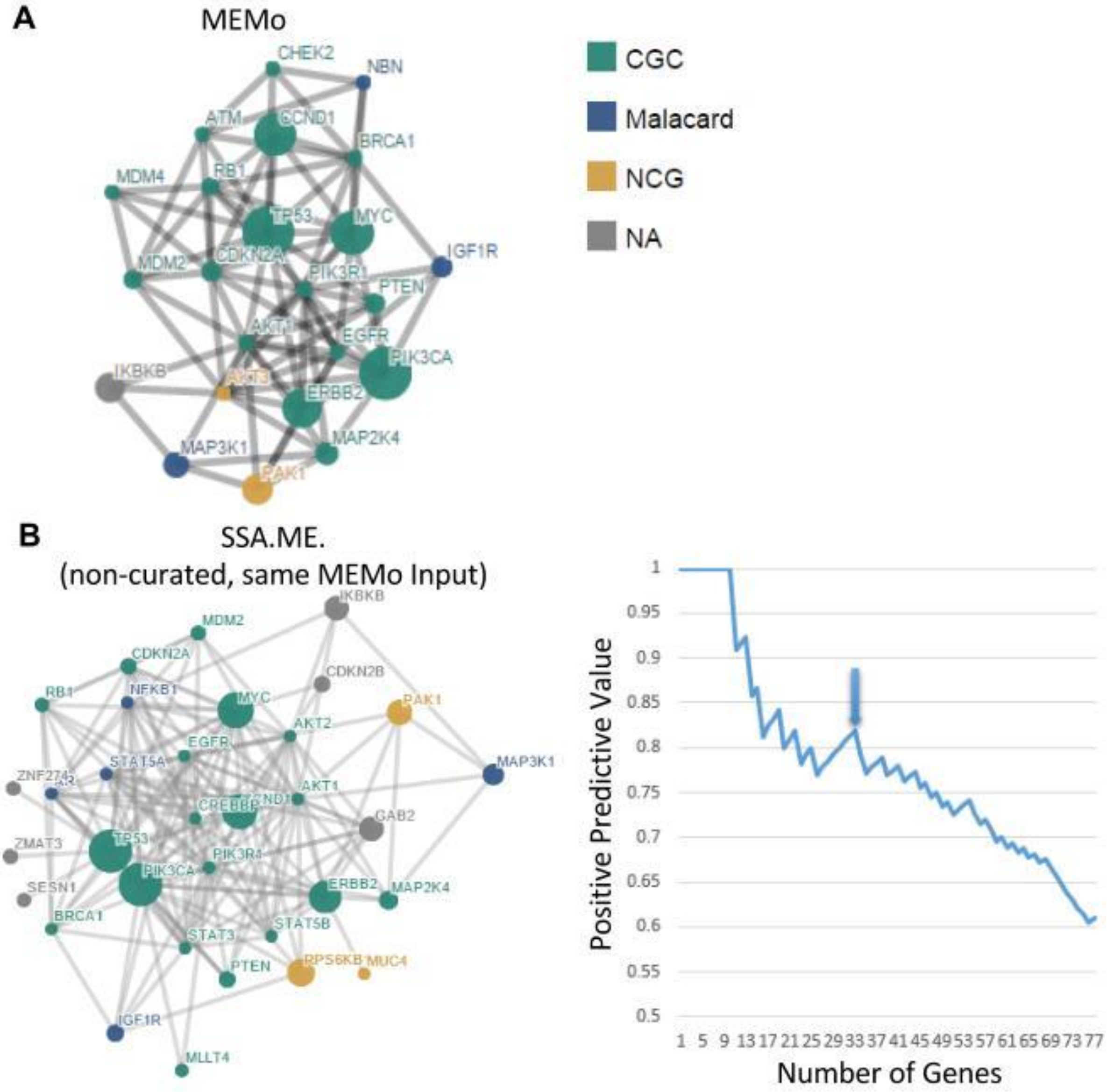

Supplementary Figure 14. Comparison between SSA-ME and MEMo. Prioritized driver networks obtained by MEMO as retrieved from the original mutually exclusive modules outlined in the breast cancer TCGA paper (Panel A) and obtained by SSA-ME using the filtered data (Panel B). Genes are represented as nodes. Colors refer to the databases in which associations of the indicated genes with breast cancer or cancer have been described. Gray genes were not found to be associated with breast cancer/cancer according to the used reference databases. The right figure in panel B represents the PPV analysis of results obtained by SSA-ME. The Y-axis represents the PPV according to the reference databases. The X-axis represents the number of genes in lists of prioritized genes of increasing order. The size of the gene list was determined by ranking the genes according to their gene scores and counting the number of genes with a rank lower than a given threshold. The Arrow indicates the thresholds that was chosen to select the genes in the network. We choose the threshold on the ranked list so that an adequate trade-off between sensitivity and precision was obtained.

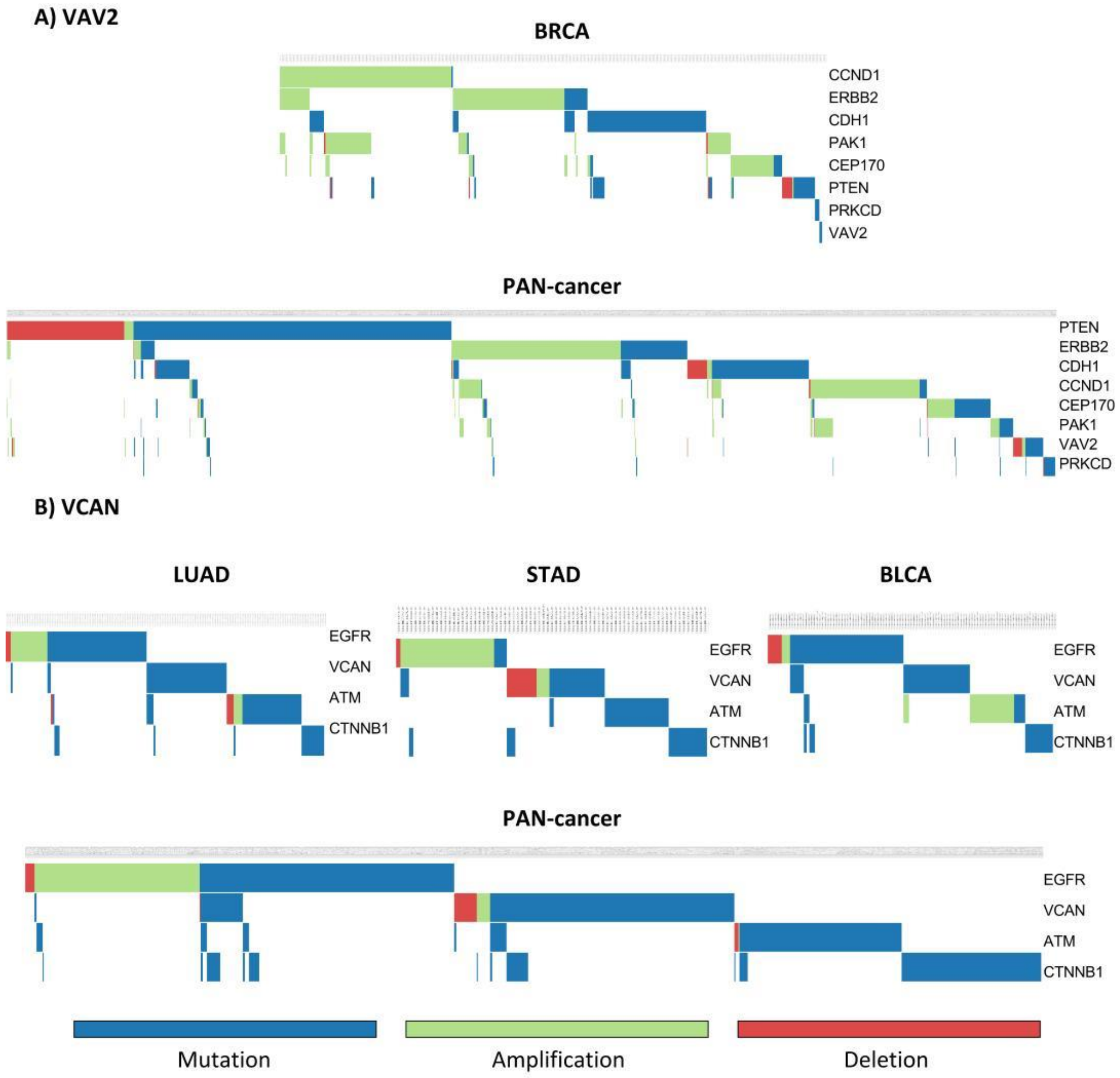

Supplementary Figure 15. Mutual exclusivity patterns of selected genes. Green tiles depict copy number gains, blue tiles depict somatic mutations and red tiles depict losses of copy number for all these patterns. A) Mutual exclusivity pattern of VAV2. The top figure visualizes the pattern in the BRCA dataset in which the pattern was originally detected. The bottom figure provides the pan-cancer view of the same pattern. TP53 and PIK3CA, which were also part of the pattern, were omitted from the visualization to allow zooming in on the less frequently mutated genes. B) Mutual exclusivity patterns of VCAN. Top panel shows the pattern in each of the three pan-cancer datasets in which the pattern was prioritized (LUAD, STAD and BLCA). The bottom figure provides the pan-cancer view of the same pattern. The genes shown correspond to the intersection of the genes present in the 5-best small subnetworks which showed highest mutual exclusivity values for each dataset in which VAV2 was prioritized (LUAD, STAD and BLCA). TP53 which was also part of the pattern but was omitted from the visualization to allow zooming in on the less frequently mutated genes.

References

1 Ruckert, F. *et al.* Examination of apoptosis signaling in pancreatic cancer by computational signal transduction analysis. *PLoS One* **5**, e12243, doi:10.1371/journal.pone.0012243 (2010).

2 Choi, S. *et al.* Bcl-xL promotes metastasis independent of its anti-apoptotic activity. *Nat Commun* **7**, 10384, doi:10.1038/ncomms10384 (2016).

3 Ertel, F., Nguyen, M., Roulston, A. & Shore, G. C. Programming cancer cells for high expression levels of Mcl1. *EMBO Rep* **14**, 328-336, doi:10.1038/embor.2013.20 (2013).
4 Hernandez, L. *et al.* Activation of NF-kappaB signaling by inhibitor of NF-kappaB kinase beta increases aggressiveness of ovarian cancer. *Cancer Res* **70**, 4005-4014, doi:10.1158/0008-5472.CAN-09-3912 (2010).
5 Kircher, M. *et al.* A general framework for estimating the relative pathogenicity of human genetic variants. *Nat Genet* **46**, 310-315, doi:10.1038/ng.2892 (2014).
6 Ding, C. B., Yu, W. N., Feng, J. H. & Luo, J. M. Structure and function of Gab2 and its role in cancer (Review). *Mol Med Rep* **12**, 4007-4014, doi:10.3892/mmr.2015.3951 (2015).
7 Bocanegra, M. *et al.* Focal amplification and oncogene dependency of GAB2 in breast cancer. *Oncogene* **29**, 774-779, doi:10.1038/onc.2009.364 (2010).
8 Bepler, G. & Koehler, A. Multiple chromosomal aberrations and 11p allelotyping in lung cancer cell lines. *Cancer Genet Cytogenet* **84**, 39-45 (1995).
9 Monni, O. *et al.* Comprehensive copy number and gene expression profiling of the 17q23 amplicon in human breast cancer. *Proc Natl Acad Sci U S A* **98**, 5711-5716, doi:10.1073/pnas.091582298 (2001).
10 Sriram, G. & Birge, R. B. Emerging roles for crk in human cancer. *Genes Cancer* **1**, 1132-1139, doi:10.1177/1947601910397188 (2010).
11 Fathers, K. E. *et al.* Crk adaptor proteins act as key signaling integrators for breast tumorigenesis. *Breast Cancer Res* **14**, R74, doi:10.1186/bcr3183 (2012).
12 Bell, E. S. & Park, M. Models of crk adaptor proteins in cancer. *Genes Cancer* **3**, 341-352, doi:10.1177/1947601912459951 (2012).
13 Zhou, X. *et al.* Nerve growth factor receptor negates the tumor suppressor p53 as a feedback regulator. *Elife* **5**, doi:10.7554/eLife.15099 (2016).
14 Civenni, G. *et al.* Human CD271-positive melanoma stem cells associated with metastasis establish tumor heterogeneity and long-term growth. *Cancer Res* **71**, 3098-3109, doi:10.1158/0008-5472.CAN-10-3997 (2011).
15 Boiko, A. D. *et al.* Human melanoma-initiating cells express neural crest nerve growth factor receptor CD271. *Nature* **466**, 133-137, doi:10.1038/nature09161 (2010).
16 Johnston, A. L. *et al.* The p75 neurotrophin receptor is a central regulator of glioma invasion. *PLoS Biol* **5**, e212, doi:10.1371/journal.pbio.0050212 (2007).
17 De Robertis, M. *et al.* Dysregulation of EGFR pathway in EphA2 cell subpopulation significantly associates with poor prognosis in colorectal cancer. *Clin Cancer Res*, doi:10.1158/1078-0432.CCR-16-0709 (2016).
18 Keppler, S. *et al.* Rare SNPs in receptor tyrosine kinases are negative outcome predictors in multiple myeloma. *Oncotarget*, doi:10.18632/oncotarget.9607 (2016).
19 Cancer Genome Atlas, N. Comprehensive molecular portraits of human breast tumours. *Nature* **490**, 61-70, doi:10.1038/nature11412 (2012).
20 Bourguignon, L. Y. *et al.* Hyaluronan promotes CD44v3-Vav2 interaction with Grb2-p185(HER2) and induces Rac1 and Ras signaling during ovarian tumor cell migration and growth. *J Biol Chem* **276**, 48679-48692, doi:10.1074/jbc.M106759200 (2001).
21 Ranzani, M., Annunziato, S., Adams, D. J. & Montini, E. Cancer gene discovery: exploiting insertional mutagenesis. *Mol Cancer Res* **11**, 1141-1158, doi:10.1158/1541-7786.MCR-13-0244 (2013).
22 Du, W. W., Yang, W. & Yee, A. J. Roles of versican in cancer biology--tumorigenesis, progression and metastasis. *Histol Histopathol* **28**, 701-713 (2013).
23 Du, W. W. *et al.* The role of versican in modulating breast cancer cell self-renewal. *Mol Cancer Res* **11**, 443-455, doi:10.1158/1541-7786.MCR-12-0461 (2013).
24 Sakko, A. J. *et al.* Versican accumulation in human prostatic fibroblast cultures is enhanced by prostate cancer cell-derived transforming growth factor beta1. *Cancer Res* **61**, 926-930 (2001).

25 Gupta, N., Khan, R., Kumar, R., Kumar, L. & Sharma, A. Versican and its associated molecules: potential diagnostic markers for multiple myeloma. *Clin Chim Acta* **442**, 119-124, doi:10.1016/j.cca.2015.01.012 (2015).

26 Fanhchaksai, K. *et al.* Host stromal versican is essential for cancer-associated fibroblast function to inhibit cancer growth. *Int J Cancer* **138**, 630-641, doi:10.1002/ijc.29804 (2016).

27 Takada, K. *et al.* Targeted disruption of the BCL9/beta-catenin complex inhibits oncogenic Wnt signaling. *Sci Transl Med* **4**, 148ra117, doi:10.1126/scitranslmed.3003808 (2012).

28 Dong, M., Pang, X., Xu, Y., Wen, F. & Zhang, Y. Ubiquitin-conjugating enzyme 9 promotes epithelial ovarian cancer cell proliferation in vitro. *Int J Mol Sci* **14**, 11061-11071, doi:10.3390/ijms140611061 (2013).

29 Yu, B. *et al.* Oncogenesis driven by the Ras/Raf pathway requires the SUMO E2 ligase Ubc9. *Proc Natl Acad Sci U S A* **112**, E1724-1733, doi:10.1073/pnas.1415569112 (2015).

30 Benard, G. *et al.* IBRDC2, an IBR-type E3 ubiquitin ligase, is a regulatory factor for Bax and apoptosis activation. *EMBO J* **29**, 1458-1471, doi:10.1038/emboj.2010.39 (2010).

31 Croft, D. *et al.* The Reactome pathway knowledgebase. *Nucleic Acids Res* **42**, D472-477, doi:10.1093/nar/gkt1102 (2014).

32 Mi, H. *et al.* PANTHER version 7: improved phylogenetic trees, orthologs and collaboration with the Gene Ontology Consortium. *Nucleic Acids Res* **38**, D204-210, doi:10.1093/nar/gkp1019 (2010).

33 Kanehisa, M. *et al.* KEGG for linking genomes to life and the environment. *Nucleic Acids Res* **36**, D480-484, doi:10.1093/nar/gkm882 (2008).

34 Yamamoto, S. *et al.* INOH: ontology-based highly structured database of signal transduction pathways. *Database (Oxford)* **2011**, bar052, doi:10.1093/database/bar052 (2011).

35 Wu, G., Feng, X. & Stein, L. A human functional protein interaction network and its application to cancer data analysis. *Genome Biol* **11**, R53, doi:10.1186/gb-2010-11-5-r53 (2010).

Table S1 Ranked genes for BRCA

GeneSymbol	CGC	NCG	Malacards
PIK3CA	TRUE	TRUE	TRUE
TP53	TRUE	TRUE	TRUE
CCND1	TRUE	TRUE	TRUE
MYC	TRUE	TRUE	TRUE
PTEN	TRUE	TRUE	TRUE
PAK1	FALSE	TRUE	FALSE
PIK3R1	TRUE	TRUE	FALSE
CDH1	TRUE	TRUE	TRUE
DDX5	TRUE	TRUE	FALSE
ERBB2	TRUE	TRUE	TRUE
RPS6KB1	FALSE	TRUE	FALSE
UFD1L	FALSE	FALSE	FALSE
RB1	TRUE	TRUE	FALSE
APC	TRUE	TRUE	FALSE
STAT3	TRUE	TRUE	TRUE
GAB2	FALSE	FALSE	FALSE
EPHA2	FALSE	FALSE	FALSE
FOXA1	TRUE	TRUE	FALSE
EGFR	TRUE	TRUE	TRUE
VAV2	FALSE	FALSE	FALSE
MAP3K1	FALSE	TRUE	TRUE
CRK	FALSE	FALSE	FALSE
BRCA1	TRUE	TRUE	TRUE
AKT1	TRUE	TRUE	TRUE
NGFR	FALSE	FALSE	FALSE
MDM2	TRUE	TRUE	TRUE
RHOA	FALSE	TRUE	FALSE
MCL1	FALSE	FALSE	FALSE
MYB	TRUE	TRUE	TRUE
ATM	TRUE	TRUE	TRUE
CDC42	FALSE	TRUE	FALSE
BCL2L1	FALSE	FALSE	FALSE
MTOR	FALSE	FALSE	TRUE
AR	FALSE	TRUE	TRUE

Table S2 Ranked genes for KIRC

GeneSymbol	CGC	NCG
VHL	TRUE	TRUE
ZNF512B	FALSE	FALSE
PBRM1	TRUE	TRUE
EPAS1	FALSE	FALSE
HIF1A	FALSE	FALSE
MDFI	FALSE	FALSE
APC	TRUE	TRUE
TCEB1	FALSE	FALSE
TCEB2	FALSE	FALSE
SQSTM1	FALSE	FALSE
FBXW11	FALSE	FALSE
SH3BP5	FALSE	FALSE
CUL2	FALSE	FALSE
VEGFA	FALSE	FALSE
ARNT	TRUE	TRUE
TP53	TRUE	TRUE
DST	FALSE	TRUE
DDX41	FALSE	FALSE
PIK3CA	TRUE	TRUE
ESR2	FALSE	FALSE
KDR	TRUE	TRUE
SYNE1	FALSE	TRUE
MTOR	FALSE	FALSE

Table S3 Ranked genes for HNSC

GeneSymbol	CGC	NCG
TP53	TRUE	TRUE
PIK3CA	TRUE	TRUE
EGFR	TRUE	TRUE
RB1	TRUE	TRUE
E2F1	FALSE	FALSE
PTEN	TRUE	TRUE
EPHA2	FALSE	FALSE
APC	TRUE	TRUE
MDM2	TRUE	TRUE
PIK3R1	TRUE	TRUE
ATM	TRUE	TRUE
CTNNB1	TRUE	TRUE
MYC	TRUE	TRUE
PPP1CA	FALSE	FALSE
DDX5	TRUE	TRUE
CHD3	FALSE	TRUE
USP7	FALSE	FALSE
CDC42	FALSE	TRUE
CDKN2A	TRUE	TRUE

Table S4 Ranked genes for STAD

GeneSymbol	CGC	NCG
TP53	TRUE	TRUE
APC	TRUE	TRUE
SMAD4	TRUE	TRUE
PIK3CA	TRUE	TRUE
EGFR	TRUE	TRUE
CTNNB1	TRUE	TRUE
ERBB2	TRUE	TRUE
SKIL	FALSE	FALSE
HDAC2	FALSE	TRUE
ATM	TRUE	TRUE
CDH1	TRUE	TRUE
PTEN	TRUE	TRUE
RNF4	FALSE	FALSE
VCAN	FALSE	TRUE
SESN1	FALSE	FALSE
HGF	FALSE	FALSE
IKBKG	FALSE	FALSE
RAC1	TRUE	FALSE
TRRAP	TRUE	TRUE
RHOA	FALSE	TRUE

Table S5 Ranked genes for GBM

GeneSymbol	CGC	NCG
CDK4	TRUE	TRUE
CDKN2B	FALSE	FALSE
CDKN2A	TRUE	TRUE
CDK6	TRUE	TRUE
MYC	TRUE	TRUE
TP53	TRUE	TRUE
MAX	TRUE	FALSE
MCM2	FALSE	FALSE
PTEN	TRUE	TRUE
MAGEA11	FALSE	FALSE
CDC5L	FALSE	FALSE
RB1	TRUE	TRUE
SMAD4	TRUE	TRUE
MDM2	TRUE	TRUE
SP1	FALSE	TRUE
PIK3R1	TRUE	TRUE
CEBPB	FALSE	FALSE
KIAA1377	FALSE	FALSE
SMAD3	FALSE	TRUE
PIK3CA	TRUE	TRUE
EGFR	TRUE	TRUE
IKBKAP	FALSE	FALSE
CDKN2C	TRUE	TRUE

Table S5 Ranked genes for GBM

GeneSymbol	CGC	NCG
CDK4	TRUE	TRUE
CDKN2B	FALSE	FALSE
CDKN2A	TRUE	TRUE
CDK6	TRUE	TRUE
MYC	TRUE	TRUE
TP53	TRUE	TRUE
MAX	TRUE	FALSE
MCM2	FALSE	FALSE
PTEN	TRUE	TRUE
MAGEA11	FALSE	FALSE
CDC5L	FALSE	FALSE
RB1	TRUE	TRUE
SMAD4	TRUE	TRUE
MDM2	TRUE	TRUE
SP1	FALSE	TRUE
PIK3R1	TRUE	TRUE
CEBPB	FALSE	FALSE
KIAA1377	FALSE	FALSE
SMAD3	FALSE	TRUE
PIK3CA	TRUE	TRUE
EGFR	TRUE	TRUE
IKBKAP	FALSE	FALSE
CDKN2C	TRUE	TRUE

Table S7 Ranked genes for UCEC

GeneSymbol	CGC	NCG
PIK3CA	TRUE	TRUE
PIK3R1	TRUE	TRUE
TP53	TRUE	TRUE
PTEN	TRUE	TRUE
MYC	TRUE	TRUE
BCL2L1	FALSE	FALSE
CTNNB1	TRUE	TRUE
ERBB2	TRUE	TRUE
CDH1	TRUE	TRUE
DNM2	TRUE	TRUE
PDGFRB	TRUE	TRUE
ERBB3	FALSE	TRUE
VAV2	FALSE	FALSE
APC	TRUE	TRUE
CTNND1	FALSE	FALSE
MAX	TRUE	FALSE
UBQLN4	FALSE	FALSE
ESR1	FALSE	FALSE
TRRAP	TRUE	TRUE
SYK	TRUE	TRUE

Table S8 Ranked genes for OV

GeneSymbol	CGC	NCG
TP53	TRUE	TRUE
MYC	TRUE	TRUE
RB1	TRUE	TRUE
MAX	TRUE	FALSE
AKT1	TRUE	TRUE
CCNE1	TRUE	TRUE
RNF144B	FALSE	FALSE
BCL2L1	FALSE	FALSE
MCL1	FALSE	FALSE
TERT	TRUE	TRUE
CCNB1	FALSE	FALSE
UBE2I	FALSE	FALSE
CTBP1	FALSE	FALSE
TAF9	FALSE	FALSE
TK1	FALSE	FALSE
SMARCA2	FALSE	FALSE
TFDP1	FALSE	FALSE
BRCA1	TRUE	TRUE
HIF1A	FALSE	FALSE
STK11	TRUE	TRUE
NFYC	FALSE	FALSE
TRIP13	FALSE	FALSE
PPP1CA	FALSE	FALSE
SFN	FALSE	FALSE
BCAT1	FALSE	FALSE
PTEN	TRUE	TRUE
APC	TRUE	TRUE
SCAMP1	FALSE	FALSE
XRCC6	FALSE	FALSE
PIK3R1	TRUE	TRUE
GSK3B	FALSE	TRUE
SKP2	FALSE	TRUE
PMS2	TRUE	TRUE
JUN	TRUE	TRUE
TRIM28	FALSE	FALSE

Table S9 Ranked genes for LUSC

GeneSymbol	CGC	NCG
TP53	TRUE	TRUE
SKP2	FALSE	TRUE
RB1	TRUE	TRUE
TRIP13	FALSE	FALSE
PTEN	TRUE	TRUE
EGFR	TRUE	TRUE
FOXM1	FALSE	FALSE
BCL2L1	FALSE	FALSE
CCNE1	TRUE	TRUE
PLK3	FALSE	FALSE
FADD	FALSE	TRUE
BID	FALSE	FALSE
TK1	FALSE	FALSE
CREB3	FALSE	FALSE
CDKN2A	TRUE	TRUE
GRB2	FALSE	TRUE
PIK3CA	TRUE	TRUE
PIK3R1	TRUE	TRUE
TFDP1	FALSE	FALSE
UFD1L	FALSE	FALSE
MDM2	TRUE	TRUE
REL	TRUE	TRUE
SETDB1	FALSE	FALSE
AKT1	TRUE	TRUE
APC	TRUE	TRUE

Table S10 Ranked genes for LUAD

GeneSymbol	CGC	NCG
TP53	TRUE	TRUE
APC	TRUE	TRUE
ATM	TRUE	TRUE
EGFR	TRUE	TRUE
CTNNB1	TRUE	TRUE
VCAN	FALSE	TRUE
STK11	TRUE	TRUE
NFYC	FALSE	FALSE
SMAD4	TRUE	TRUE
MET	TRUE	TRUE
ERBB2	TRUE	TRUE
SIK1	FALSE	FALSE
SMARCA4	TRUE	TRUE
TFDP1	FALSE	FALSE
USP7	FALSE	FALSE
MAX	TRUE	FALSE
HGF	FALSE	FALSE
DST	FALSE	TRUE
CASP3	FALSE	FALSE
RAC1	TRUE	FALSE
LAMA4	FALSE	TRUE
BCL2L1	FALSE	FALSE
MACF1	FALSE	TRUE
BRCA1	TRUE	TRUE
BCL6	TRUE	TRUE
CDC42	FALSE	TRUE

Table S11 Ranked genes for BLCA

GeneSymbol	CGC	NCG
TP53	TRUE	TRUE
MDM2	TRUE	TRUE
RB1	TRUE	TRUE
MYC	TRUE	TRUE
PIK3CA	TRUE	TRUE
ATM	TRUE	TRUE
CDKN1A	FALSE	FALSE
CCND1	TRUE	TRUE
APC	TRUE	TRUE
BCL2L1	FALSE	FALSE
CREBBP	TRUE	TRUE
DDX5	TRUE	TRUE
USP7	FALSE	FALSE
CTNNB1	TRUE	TRUE
ERBB2	TRUE	TRUE
PPARG	TRUE	TRUE
BNIP3L	FALSE	FALSE
HDAC2	FALSE	TRUE
EWSR1	TRUE	TRUE
TK1	FALSE	FALSE
TRRAP	TRUE	TRUE
BRCA1	TRUE	TRUE
YWHAZ	FALSE	TRUE
CDKN2A	TRUE	TRUE
EGFR	TRUE	TRUE

Table S12 Ranked genes for LAML

GeneSymbol	CGC	NCG
TP53	TRUE	TRUE
DNMT3A	TRUE	TRUE
MYC	TRUE	TRUE
MAX	TRUE	FALSE
ZHX1	FALSE	FALSE
PTPN11	TRUE	TRUE
NRAS	TRUE	TRUE
IDH1	TRUE	TRUE
KIT	TRUE	TRUE
HNRNPK	FALSE	FALSE
